# Establishing normative data for the evaluation of cognitive performance in Huntington’s disease considering the impact of gender, age, language, and education

**DOI:** 10.1007/s00415-023-11823-x

**Published:** 2023-06-22

**Authors:** Alžbeta Mühlbäck, Josef Mana, Michael Wallner, Wiebke Frank, Katrin S. Lindenberg, Rainer Hoffmann, Olga Klempířová, Jiří Klempíř, G. Bernhard Landwehrmeyer, Ondrej Bezdicek, the REGISTRY investigators of the European Huntington’s Disease Network, the Enroll-HD investigators

**Affiliations:** 1 Department of Neurology, Ulm University, Oberer Eselsberg 45, 89081 Ulm, Germany; 2 Huntington Center South, kbo-Isar-Amper-Klinikum, Taufkirchen, Germany; 3 Department of Neurology and Center of Clinical Neuroscience, 1st Faculty of Medicine, Charles University and General University Hospital in Prague, Prague, Czechia; 4 2mt Software GmbH, Ulm, Germany

**Keywords:** Huntington’s disease, Cognition, Cognitive decline, Neuropsychological testing, Normative data, Online calculator

## Abstract

**Background:**

A declining cognitive performance is a hallmark of Huntington’s disease (HD). The neuropsychological battery of the Unified HD Rating Scale (UHDRS'99) is commonly used for assessing cognition. However, there is a need to identify and minimize the impact of confounding factors, such as language, gender, age, and education level on cognitive decline.

**Objectives:**

Aim is to provide appropriate, normative data to allow clinicians to identify disease-associated cognitive decline in diverse HD populations by compensating for the impact of confounding factors

**Methods:**

Sample data, *N* = 3267 (60.5% females; mean age of 46.9 years (*SD* = 14.61, range 18–86) of healthy controls were used to create a normative dataset. For each neuropsychological test, a Bayesian *generalized additive model* with age, education, gender, and language as predictors was constructed to appropriately stratify the normative dataset.

**Results:**

With advancing age, there was a non-linear decline in cognitive performance. In addition, performance was dependent on educational levels and language in all tests. Gender had a more limited impact. Standardized scores have been calculated to ease the interpretation of an individual’s test outcome. A web-based online tool has been created to provide free access to normative data.

**Conclusion:**

For defined neuropsychological tests, the impact of gender, age, education, and language as factors confounding disease-associated cognitive decline can be minimized at the level of a single patient examination.

**Supplementary Information:**

The online version contains supplementary material available at 10.1007/s00415-023-11823-x.

## Introduction

HD is an autosomal-dominant, progressive neurodegenerative disorder caused by an expansion of a trinucleotide cytosine-adenine-guanine (CAG) repeat in the huntingtin gene (*htt)* [1] impairing motor and cognitive performance and disrupting behavior [2]. The clinical diagnosis of HD is typically based on the emergence of characteristic extra-pyramidal motor signs combined with a molecular genetic test indicating an expansion of the CAG repeat into the pathological range [2]. Numerous studies established (1) that performance decline in tests assessing psychomotor speed is an early, well-reproduced feature of HD [3–5] and (2) that performance decline in the neuropsychological tests, in general, is—along with an increasing motor impairment—a consistent, quantifiable hallmark of disease progression in HD [2, 6]. However, several confounding factors complicate the interpretation of a decline in cognitive performance scores in HD patients, with age-related deterioration playing an important role. This is particularly impactful in the prodromal stages of HD with ‘normal’ decline, not reflecting HD-associated neurodegeneration. Further, it is of critical importance for clinical purposes to isolate the impact of disease progression on cognitive performance from confounding factors, such as age [7], gender [8], level of education [9], language and cultural background [10] at a single-subject level.

In HD, for tracking disease progression, a battery of cognitive tests well suited for repeated application is commonly used [6, 11]. Although there are several cognitive tests available to assess cognitive decline in HD, there is currently no gold standard for assessing cognition [12]. Most HD centres rely on the cognitive battery that is part of the United Huntington Disease Rating Scale (UHRDS) [13] and of the observational cohort study Enroll-HD in daily clinical practice [6, 14]. The Enroll-HD cognitive battery examined in this study includes the Symbol Digit Modalities Test (SDMT) [15], the Stroop Tests consisting of the Stroop Word Reading Test (SWRT), Stroop Color Naming Test (SCNT) and Stroop Interference Test (SIT) [16, 17] as well as the Trail Making Test-Part A (TMT-A) und Part B (TMT-B) [18, 19], and the Letter Fluency Test (LFT) [20], and Category Fluency Test (CFT) [20].

It is worth to highlight that several components of the Enroll-HD cognitive test battery differ from the original test version and mode of application described in the respective foundational publications. E.g., Stroop described initially using the five colors red, blue, green, brown and purple and used time for completion as the raw score [16]. Golden changed the SWRT, SCNT, SIT matrix of the current standardized version the number of colors from five to three, with 100 items (words/colors) printed per card and stopping probands after 45 s, using the number of correct answers as the raw score [17]. No normative data for this particular mode of application can be derived from the original publication; only scattered normative data, restricted to a few languages, are published [21].

Overall, no comprehensive, recent set of normative data for the particular mode of application of these commonly employed cognitive tests stratified by gender, age, language and education are available.

## Methods

In this project, data collected in the context of the prospective cohort study Enroll-HD (ClinicalTrials.gov Identifier: NCT01574053) were used. Core phenotypic clinical datasets are collected annually from all research participants, including controls, as part of a multicentre, prospective longitudinal observational study. Enroll-HD was approved by the local institutional ethical review boards at every study site and conducted in accordance with the ethical standards described in the Declaration of Helsinki of 1964. All participants provided written informed consent for their participation. Any information that could risk disclosing the identity of the participants was omitted.

### Participants—sample selection

The data sample (healthy control population) was extracted from the periodic data set 4 (PDS4) of the Enroll-HD study, which includes 15,301 participants from 139 study sites across 3 continents [22]. Enroll-HD contains data from two groups of participants: (1) participants carrying the *htt-*expansion mutation, either at clinically manifest or at premanifest stage and (2) participants known not to carry th*e htt* expansion mutation, i.e., HD family members who opted for predictive genetic testing and learnt that they are genotype-negative as well as family controls (partners, caregivers) who were shown to carry a CAG repeat expansion within the physiological range at the *htt*-gene by undisclosed genotyping.

**Fig. 1:**
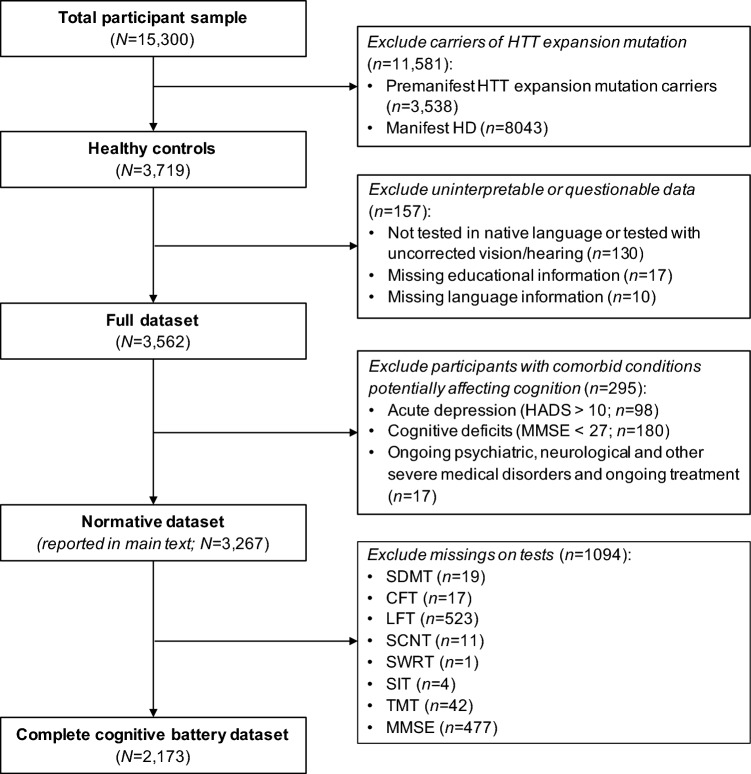
Flow chart depicting the selection process for a normative dataset as reported in the main text. Results from the complete cognitive battery dataset (*N* = 1173) and the full dataset (*N* = 3562) can be provided at the request of the corresponding author

For the normative study, we only included healthy controls, i.e., participants without a comorbid conditions that likely influences cognitive performance, who fulfilled additional predefined inclusion and exclusion criteria (see Fig. 1).

The study used a cross-sectional design, meaning that the first baseline neuropsychological assessment after enrollment in the Enroll-HD study was used to avoid practice/learning effects in the normative population. Participants who were not examined in their native language or examined with uncorrected vision or hearing were excluded, as were participants for whom educational or language information was missing. To avoid an adverse impact of depression on cognitive functioning, participants with evidence for ongoing depression at the time of the cognitive assessment using the Hospital Anxiety Depression Scale (HADS) [23] with a score > 10 were removed from the sample. In addition, participants were screened for cognitive impairment by the Mini-Mental Status Examination (MMSE) [24], and data from those with MMSE score ≤ 26 were not considered.

### Neuropsychological assessment

All participants were examined by trained personnel (i.e., clinicians, psychologists, study nurses) using the standardized neuropsychological assessment protocol as a part of the Enroll-HD cohort study [25] including SDMT [15], SCNT, SWRT, SIT [17] with modified application rules, TMT-A and TMT-B [18], LFT and VFT [20], and MMSE [24].

The MMSE is a screening questionnaire consisting of 11 items to measure general cognitive functioning applied as per the standard operating procedure of the original publication [24].

The SDMT is a timed psychomotor test in which participants have to match numbers to symbols within 90 s, by writing down numbers as motor response [15].

The Stroop test battery is composed of three sub-tests, the SWRT, SCNT, SIT. The first two sub-tests, the SWRT and SCNT, measure attention and psychomotor speed. The participants have to name the color of ink patches (SCNT) and read words indicating colors (red, blue, green) printed in black ink (SWRT). The third subtest is used as a measure for response inhibition [17]. The participants see words indicating colors (red, blue, green), each written in red, blue or green ink, incongruent to the color indicated by the letters (e.g., the word 'red' written in blue ink). Probands have to inhibit uttering the words read and instead have to name the color of the ink in which the incongruent color word is printed. The color names uttered are recorded by the examiner, and the total score indicates the number of correct responses in 45 s.

In the LFT, participants are requested to utter as many words as possible, starting with a particular letter within 1 min (response is speech motor output). LFT consists of three sub-tests, each using a different letter (i.e., F, A, S in English), tapping into the categories of high, medium, and low frequency in the lexicon of the respective language [26], the total score reported is the sum of all sub-tests' correct, unique words. CFT resembles LFT and requires participants to verbally produce as many words as possible within the semantic category, e.g., ‘animal’ in 1 min [20].

The TMT consists of two tasks with drawing straight lines connecting appropriate circles as motor output [19]. The instructions for connecting circles labeled with numbers or letters differ between the two tasks: in TMT-A, participants have to connect given numbers in ascending order (i.e., 1-2-3-4); in TMT-B, numbers and letters in alternating order (i.e., 1-A-2-B-3-C;).

### Statistical analyses

Descriptive statistical analyses were applied to describe the number of participants and their demographic characteristics. Continuous variables were characterized by means (*M*), standard deviations (*SD*), medians, and ranges. Categorical variables were expressed as percentages to the whole sample. Table norms (as showed in Supplementary Material Tables 4–10) consisting of *M* and *SD* stratified by age, an education level (≤ 12 years and > 12 years of education), gender and language were constructed when feasible. Education years were dichotomized for the table norms to establish a lower (≤ 12 years) and higher (> 12 years) educated group of participants. This dichotomization allows us to consider the differences between education levels while keeping the norms easy to read; a similar approach has been used to create norms in Alzheimer’s disease [27] and HD [28]. However, in the regression-based norms, education in years, age, gender, and language were used as confounding variables.

### Development of regression-based norms

To develop the regression-based norms, a normative probability mapping approach extended to behavioral data [29–31] was employed.

This approach uses Bayesian modeling to estimate a normative distribution of an outcome of interest (e.g., a cognitive test) at different levels of relevant demographic variables (e.g., age and years of education). The normative distribution is used to predict an outcome of a new individual based on their demographic characteristics.

In line with Marquand, Rezek [29] and Wang, Herrington [30], we applied a method of normative probability mapping to construct normative *z* scores for each of the included cognitive measures.

Since TMT-A and TMT-B are reverse coded, whereby lower scores indicate better performance, *z* scores for these two measures were multiplied by minus one to keep interpretation consistent with the rest of the measures.

To select an appropriate model for regression-norms development, several candidate models for each cognitive outcome were considered: (i) linear multiple regressions (LMRs) with age, years of education, language and gender as predictors and (ii) generalized additive models (GAMs) with the same predictors, however, using tensor splines to model possibly non-linear additive effects of age and years of education [32]. To evaluate the predictive performance of each demographic variable, models were fitted using either all four predictors or dropping one predictor at a time. These models were subsequently compared via leave-one-out information criterion (LOO-IC) [33], an approximate measure of expected predictive accuracy.

### Analysis of cognitive differences according to gender and language

Differences in gender and language were tested using the models, which were estimated to develop the regression-based norms. In particular, gender differences in cognitive performance were evaluated by examining the posterior parameter distributions of models that included all predictors, i.e., age, years of education, gender, and language. The differences were reported such that positive values indicated higher scores for women than for men and vice versa.

A two-step analysis procedure was carried out to evaluate differences in cognitive performance across the different languages. First, models containing all predictors were compared to models containing age, years of education, and gender only via leave-one-out information criterion LOO-IC [33], a measure of expected predictive accuracy. The differences in LOO-IC were described by their mean and 95% confidence intervals (CI). A difference was considered significant if the 95% CI did not contain zero. If the LOO-IC model comparison showed that the full model offers better predictive performance, the parameter estimates of the full models were examined in the second step. Model parameters were described by their means and 95% highest density posterior probability intervals (PPI), i.e., the shortest interval with a 95% probability of containing the true parameter value.

All models were estimated using the Stan software version 2.19 [34] accessed via the brms package [35] in R language [36]. Due to their positive skewness, TMT-A and TMT-B were log-transformed prior to the inclusion in the analysis. Each model was estimated using a Hamiltonian Monte Carlo sampling algorithm with four chains consisting of 2000 iterations, out of which 1000 iterations were discarded as a warm-up resulting in the final sample of 4000 draws from the posterior distribution. All models were estimated using average likelihood and non-informative improper flat priors over predictor parameters. Weakly informative priors for model intercepts and parameters’ standard deviations were used to ensure convergence. The quality of the sampling algorithm was checked numerically by inspection of the potential scale reduction factor (R̂s) and visually by inspection of trace plots and posterior predictive probability plots [37]. The results were screened for influential observations using Pareto k̂ statistic [38].

## Results

### Demographic characteristics and overall cognitive performance of the normative sample

In total, the normative sample included a cohort of *N* = 3267 (1978 females, 1289 males) healthy controls with mean age of *M* = 46.99 years (*SD* = 14.61, ranging from 18 to 86) and an average number of years of education *M* = 14.66 (*SD* = 3.27, ranging from 1 to 24). The assessments were administered in the following languages (in descending order of frequency): English, German, Spanish, Italian, Polish, French-Canadian, French, Dutch, Spanish Latin-American. Demographic characteristics of the normative sample are presented in Table 1. Descriptive statistics on the performance of the normative sample (*N* = 3267) in the neuropsychological tests are presented in the lower half of Table 1.

**Table 1:** Demographic characteristics and descriptive statistics on the performance on neuropsychological assessments of the normative sample (NS; *N* = 3267)

Age* (in years)				*N* 3267	*M* 46.99		SD 14.61	Median 48		Range 18–86
Age Groups	18–25	25–29	30–34	35–39	40–44	45–49	50–54	55–59	60–64	> 64
*N* (%)	209 (6.4)	256 (7.8)	323 (9.9)	321 (9.8)	310 (9.5)	344 (10.5)	396 (12.1)	368 (11.3)	323 (9.9)	417 (12.8)

Gender*	Female	Male
*N* (%)	1978 (60.5)	1289 (39.5)

Education levels	ISCED 0	ISCED 1	ISCED 2	ISCED 3	ISCED 4	ISCED 5	ISCED 6
*N* (%)	5 (0.1)	76 (2.3)	273 (8.4)	890 (27.2)	704(21.6)	1227 (37.6)	92 (2.8)

Years of education*	*N*	*M*	*SD*	Median	Range
(in years)	3267	14.66	3.27	14	1–24

Education years	≤ 12 years	> 12 years
*N* (%)	852 (26.1)	2415 (73.9)

Laterality	Right-handed	Left-handed	Mixed
*N* (%)	2933 (89.8)	253 (7.7)	81 (2.5)

Language	English	German	Spanish	Italian	Polish	Canadian French	French	Dutch	Latino Spanish	Danish
*N* (%)	1808 (55.3)	639 (19.6)	299 (9.2)	238 (7.3)	98 (3.0)	70 (2.1)	21 (0.6)	46 (1.4)	29 (0.9)	19 (0.6)

Ethnicity	Caucasian	American Black	Hispano or Latino Origin	American Indian/Native American	Asian	Mixed	Other
*N* (%)	3031 (92.8)	28 (0.9)	81 (2.5)	18 (0.6)	19 (0.6)	44 (1.4)	46 (1.4)

Cognitive performance measures	*N*	*M*	*SD*	Median	Range
Symbol Digit Modalities Test	3248	50.7	11.7	51.0	0–101
Category Fluency Test	3243	22.2	5.5	22.0	3–48
Stroop Color Naming Test	3237	75.4	14.1	75.0	0–140
Stroop Word Reading Test	3242	96.4	17.1	98.0	2–168
Stroop Interference Test	3059	43.2	11.2	43.0	0–119
Trail Making Test, Part A Test	2759	27.1	15.1	24.0	8–240
Trail Making Test, Part B	2754	57.6	32.7	49.0	16–240
Letter Fluency Test	2726	41.6	12.4	41.0	7–94
Mini-Mental Status Examination	2355	29.3	0.9	30.0	27–30

*N* number of participants, *M* mean, *SD* standard deviation, *ISCED* International Standard Classification of Education, *ISCED 0* early childhood education, *ISCED 1* primary education, *ISCED 2* lower secondary education, *ISCED 3* upper secondary education, *ISCED 4* post-secondary education, *ISCED 5* short-cycle tertiary education, *ISCED 6* bachelor’s degree or equivalent level

*Variables that were used as predictors for the regression-based norms

### Regression-based normative values

All models converged in a specified number of iterations (${\hat{\text{R}}\text{s}}$  < 1.01), and there were no influential observations (Pareto ${\hat{\text{k}}\text{s}}$  < 0.5). For all tests, GAMs outperformed LMRs (see Supplementary Material Table 1). GAM model containing all variables had best predictive performance in case of SDMT, SCNT, TMT-A, and LFT, while model excluding gender had best predictive performance in CFT, SWRT, SIT, and TMT-B. According to the posterior predictive check, models’ predictions corresponded well with observed data (see Supplementary Material Fig. 1).

### Age-related differences in cognitive performance

**Fig. 2:**
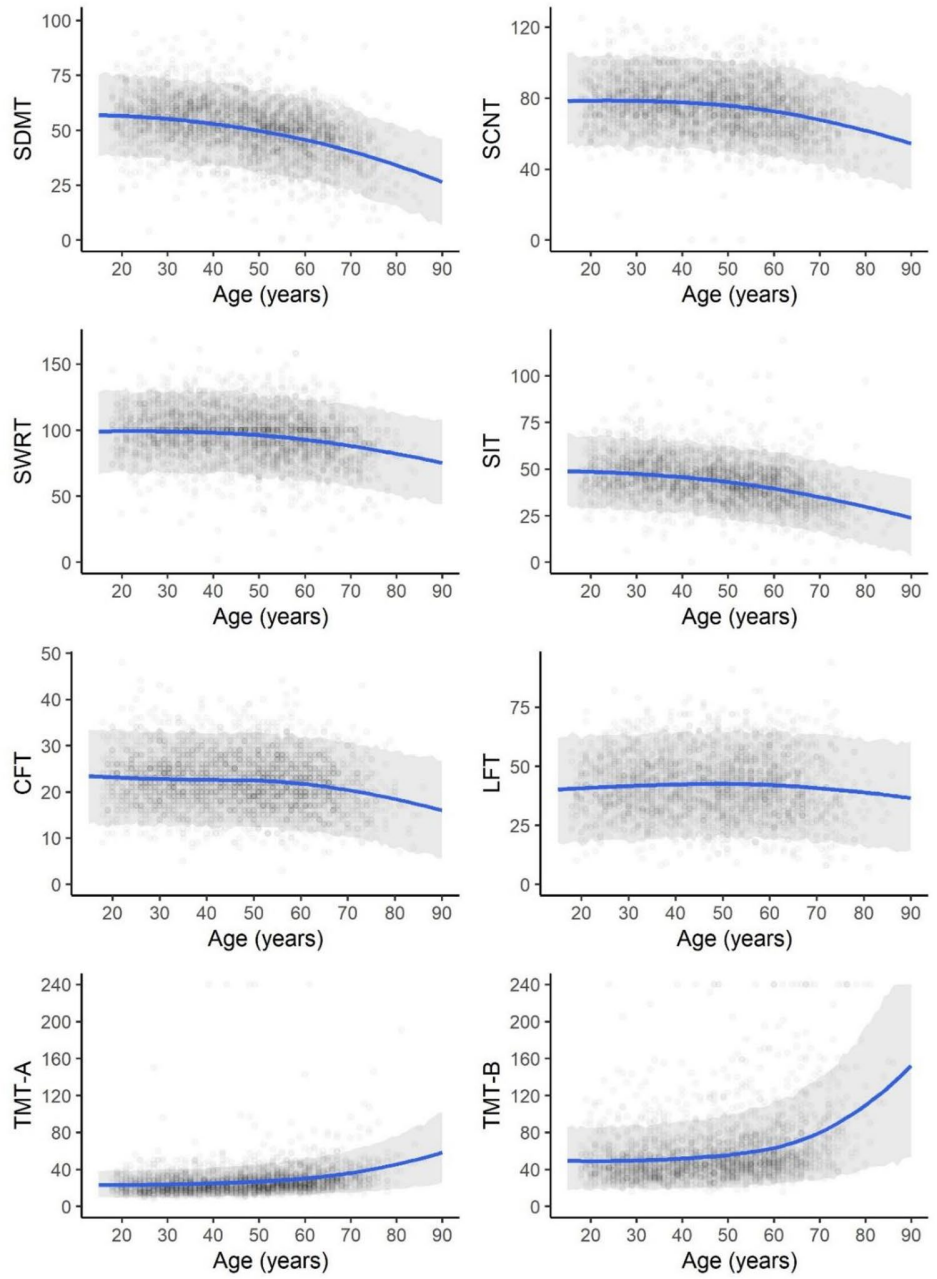
Posterior predictive plot depicting the relationship between age and performance in cognitive tests: blue lines represent the expected cognitive performance in raw values for different years of age under the constraint of assuming 14 years of formal education while refraining from specifying gender and language used during the examination; grey bands represent 95% posterior predictive intervals, and dots represent observed values

The relationship between age and cognitive tests is presented in Fig. 2 and Supplementary Material Table 2. There was a non-linear decline in performance with advancing age for all cognitive tests when adjusting for years of education, gender, and language. For CFT and LFT, a shallow decline was found. We observed an accentuated age-related decline in cognitive performance in all tests, with the decrease in performance being more pronounced in later decades than in earlier decades. Not only the TMT-A, also, particularly the TMT-B are markedly influenced by increasing age.

### Education-related differences in cognitive performance

**Fig. 3:**
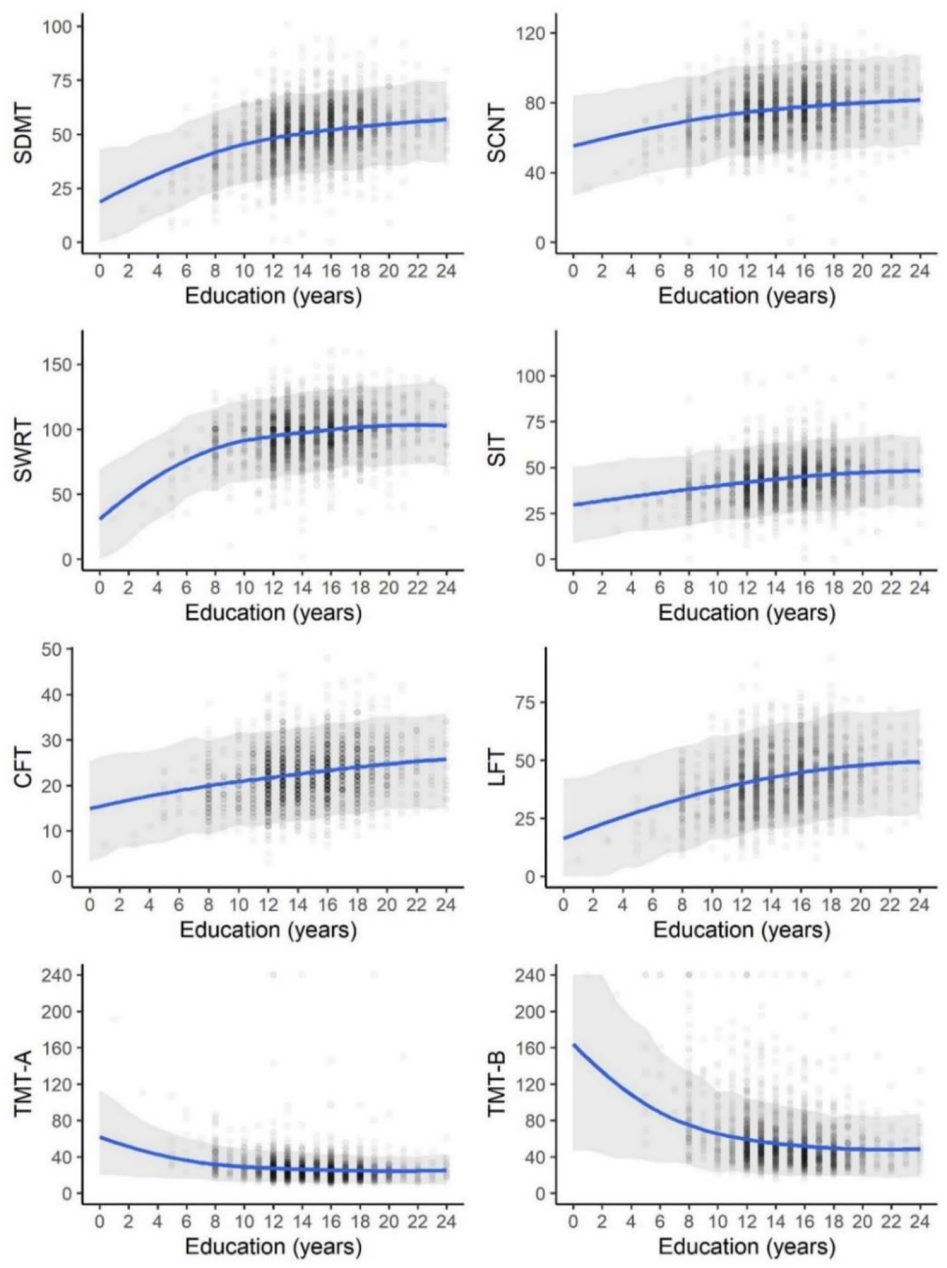
A posterior predictive plot of the relationship between years of formal education and performance in the cognitive test (raw values): blue lines represent the expected cognitive performance for different years of formal education under the constraint of assuming a participant of 48 years of age while refraining from specifying gender and language; grey bands represent 95% posterior predictive intervals, and dots represent observed values

The association between education and cognitive performance is presented in Fig. 3 and Supplementary Material Table 3. We observed a positive correlation between performance and years of formal education for all cognitive tests. More extended education level was associated with better cognitive performance when adjusting for age, gender, and language. Non-linearity was marked for the relationship between years of education and cognitive performance in all tests, with a smaller impact as years of education increase. For all tests, non-linearity was observed for the association between years of education and cognitive performance, with the effects becoming narrower by increasing years of education. SIT seems to be the least dependent on the years of education, in contrast to LFT, which shows a stronger effect.

### Gender-related differences in cognitive performance

Concerning the impact of gender on cognitive performance, our data when adjusting for years of education, gender, and language indicate that females performed significantly better than males in SDMT (*M* = 2.78, 95% PPI [2.07, 3.44]). Additionally, females displayed a slightly better performance than males in SCNT (*M* = 0.94, 95% PPI [0.03, 2.00]) and LFT (*M* = 1.33, 95% PPI [0.41, 2.20]), while males performed slightly better than females in TMT-A (*M* = 1.02, 95% PPI [1.00, 1.05]) and TMT-B (*M* = 0.98, 95% PPI [0.95, 1.01]). No clear gender-related differences were observed in CFT (*M* = 0.32, 95% PPI [− 0.09, 0.69]), SWRT (*M* = 0.59, 95% PPI [− 0.51, 1.81]), and SIT (*M* = 0.50, 95% PPI [− 0.19, 1.28]).

### Language-related differences in cognitive performance

There were language-dependent differences in cognitive performance for all cognitive tests after adjusting for years of education, gender, and language. This is demonstrated by the fact that the full models (including language as predictor) were significantly better in terms of the LOO-IC comparisons than the reduced models, which only included age, years of education, and gender as predictors (LOO-IC differences ranged from − 162.39 to − 32.97, *SE*s = 13.94–28.38, all 95% CIs excluded zero). The most extensive data set obtained from English native speakers was used as a reference for the comparison between performance data from native speakers grouped by language. The mean expected performance on all cognitive tests for each language group and their 95% PPIs is depicted in Supplementary Material Table 2. In addition, all language-dependent differences in the cognitive performance for all cognitive tests are listed in detail in Table 2. Given the low numbers of normal controls for Danish, Dutch, French, and Spanish Latin-America (< 50), we focus on apparent differences between English, German, Spanish, Italian, Polish, and French-Canadian language groups. There were differences in performance even in the tests considered “language-independent”, such as SDMT and TMT-A and B. The Spanish, Italian, and Polish native speakers, performed worse than French-Canadian and German native speakers, who performed similarly to English native speakers in TMT-A and B. The performance in SDMT in German native speakers was worse than the one observed in English native speakers but better than Polish and Italian native speakers.

**Table 2:** Differences in cognitive performance stratified by language

Language	Symbol Digit Modalities Test	Category Fluency Test	Stroop Color Naming Test	Stroop Word Reading Test	Stroop Interference Test	Trail Making Test-A	Trail Making Test -B	Letter Fluency Test
English	0	0	0	0	0	0	0	0
German	− 2.86 [− 3.77, − 2.03]	0.94 [0.45,1.41]	− 3.03 [− 4.22, − 1.85]	2.95 [1.43, 0.37]	− 1.87 [− 2.83, − 0.96]	0.05 [0.02, 0.08]	0.05 [0.01, 0.09]	− 4.19 [− 5.37, − 3.05]
Spanish	− 2.14 [− 3.38, − 0.88]	0.07 [− 0.61,0.75]	− 1.70 [− 3.43, 0.02]	7.28 [5.25, 9.39]	1.25 [− 0.12, 2.61]	0.18 [0.14, 0.23]	0.21 [0.16, 0.26]	− 2.46 [− 4.03, − 0.88]
French	2.46 [− 1.70, 6.59]	1.04 [− 1.13,3.40]	7.97 [2.79, 13.62]	6.97 [− 0.12,14.01]	2.62 [− 1.49, 6.85]	− 0.05 [− 0.20,0.10]	− 0.09 [− 0.26,0.08]	5.09 [0.09, 10.09]
Canadian French	0.39 [− 1.87, 2.64]	− 1.50 [− 2.76, − 0.28]	2.06 [− 1.16, 5.11]	2.14 [− 2.00, 5.69]	− 0.13 [− 2.43, 2.25]	− 0.02 [− 0.11,0.06]	− 0.08 [− 0.17,0.02]	− 0.29 [− 3.27, 2.69]
Dutch	− 0.05 [− 2.84, 2.85]	2.71 [1.18, 4.33]	1.35 [− 2.50, 5.08]	2.97 [− 1.91, 7.37]	2.10 [− 1.10, 4.89]	− 0.10 [− 0.20,0.00]	− 0.11 [− 0.22,0.00]	− 5.06 [− 8.61, − 1.61]
Italian	− 6.29 [− 7.58, − 4.90]	− 0.88 [− 1.65, − 0.11]	− 4.19 [− 6.03, − 2.24]	1.64 [− 0.64, 3.94]	− 1.66 [− 3.15, − 0.14]	0.24 [0.18, 0.29]	0.27 [0.20, 0.33]	− 2.49 [− 4.40, − 0.62]
Latin-America	− 6.57 [− 9.71, − 2.83]	− 2.19 [− 4.20, − 0.13]	− 8.15 [− 12.93, − 3.61]	− 3.91 [− 9.55, 1.97]	− 2.51 [− 6.41, 1.36]	0.41 [0.28, 0.54]	0.44 [0.29, 0.59]	− 0.38 [− 5.14, 4.10]
Polish	− 6.00 [− 7.97, − 4.12]	− 0.17 [− 1.19, 0.93]	− 7.02 [− 9.64, − 4.03]	− 7.80 [− 11.03, − 4.47]	− 4.78 [− 6.85, − 2.66]	0.24 [0.17, 0.31]	0.23 [0.15, 0.31]	1.21 [− 1.13, 3.62]
Danish	0.82 [− 3.39, 5.00]	3.00 [0.73, 5.43]	− 0.83 [− 6.49, 4.91]	− 4.38 [− 11.30, 3.07]	0.26 [− 4.47, 4.77]	− 0.12 [− 0.29,0.03]	− 0.01 [− 0.18,0.17]	3.01 [− 2.25, 8.36]

Values represent posterior means and their 95% posterior probability intervals (PPI, in brackets); posteriors of non-English languages were compared to English as a reference category; consequently, negative values indicate lower score, positive values indicate higher score than the one observed with English speaking participants

The differences between native speakers in performance in SDMT and TMT remained significant even after adjustment for age and educational levels between the various groups of native speakers. In language-dependent tasks like the SWRT, clear differences between groups of native speakers were apparent: native Spanish speakers excelled in SWRT, whereas speakers of Polish appeared to perform worse than all other language groups in all three sub-tests of Stroop. In contrast, performance in the CFT and LFT of Polish native speakers was like English native speakers. However, German and Spanish native speakers (and to a lesser extent native Italian speakers) appeared to perform worse in the LFT.

Performing the same analyses on both the full dataset (*N* = 3562) and on the more restricted complete cognitive battery dataset (*N* = 2173; which includes only participants who completed all cognitive tests) showed equivalent results (see Fig. 1 for a definition of the different datasets).

### Normative calculator

Our statistical model is able to produce expected standardized outcomes for each combination of input values. While this would, for its sheer size, not be manageable as table norms, it can be stored within a database. Therefore, an interactive “normative calculator” tool was created as a server-based web application to enable accurate time-efficient evaluation of patients’ cognitive performance in daily clinical practice. The tool uses look-up tables containing every possible combination of values for test and stratifying variables (age, years of education, gender, language) to transform the measured test values into *z* scores All researchers and clinicians can freely access and use the normative calculator on the following website: http://nc.2mt-software.de. After entering a patient’s demographic data and measured test values for each neuropsychological test, the calculator automatically provides the corresponding *z* scores and percentiles. The calculator assigns a color code based on the given percentile to allow for an unequivocal interpretation of each cognitive score Screenshots of the online tool are available as Supplementary Material Fig. 3.

## Discussion

Several studies have highlighted the importance of monitoring the longitudinally cognitive decline in carriers of the *htt* expansion mutation at early stages of the disease process (clinically premanifest HD) in determining the progression of the disease [3, 39–41]. However, we identified several factors impacting test performance in healthy controls, including gender, age, level of education, and language.

Stratification or conversion of raw values into *z* scores is required, to reduce the impact of these confounders and to isolate disease-associated alterations. Using standardized performance scores controlled for the influence of identified confounding factors instead of raw values helps to define and quantify the ‘real’, i.e., disease-associated alterations in cognitive performance assisting interpretation of test results in a meaningful way [42]: a measured test value can be considered “normal” if the corresponding *z* score is not more than one standard deviation away from zero, i.e., between − 1 and 1. While it is, for example, with no further information at hand, unclear how to classify a measured SDMT score of 60 symbols, the corresponding *z* score of 0.64 tells that the score is slightly above average, though still within the normal range.

Our normative study showed that increasing age is associated with a declining performance in most cognitive tests involving processing speed and executive functions, well-aligned with prior reports [43–45]. An exception is, to some extent, performance in LFT [46]. The more limited impact of age on LFT performance is in line with previous studies [47, 48] and may reflect that cognitive functions relying on crystallized intelligence are less sensitive to age-related decline [49].

As expected, higher levels of education were associated with better performance in all cognitive tests [28, 50, 51]. In addition, we observed gender effects: there was a trend toward better performance in females, most marked in SDMT. Performance in the Stroop tests and verbal fluency measures are not influenced by gender, in line with previous normative studies [46, 52].

A novel finding was the extent to which the language in which the tests are administered influences the cognitive performance. This observation is consistent with previous studies providing evidence for the impact of language [52, 53] and culture [54] on performance in neuropsychological tests, emphasizing the need to address cultural and language biases on cognitive testing [55].

Our survey also allows for a quantification of the impact of language as can be seen in Table 2: In SWRT, for example, a Polish native speaker would—in average—be expected to score 7.80 points worse than an English native speaker. The performance of a (fictional) 34-year-old female Polish native speaker with 13 years of education and a SWRT raw score of 77 can, therefore, still be considered normal (*z* score: − 0.68). A 34-year-old female English native speaker with 13 years of education and the same raw score of 77 would be considered below average (*z* score: − 1.24). To compare both scores, the “Polish” raw score would have to be shifted by 7.80 with the resulting “English” score of 77 + 7.80 ≈ 84 providing the expected *z* score of − 0.68. The normative calculator tool can be used to provide more accurate results compared to the averaged values (see also Supplementary Material Fig. 4).

Overall, we established the first comprehensive set of normative data for the Enroll-HD cognitive test battery stratified by gender, age, language, and education.

### Strengths and limitations

Among the strengths of this study are a relatively large overall number of control subjects with prospectively and systematically obtained cognitive assessments in bona fide ‘healthy’ subjects, prospectively screened for comorbid conditions. As the Enroll-HD platform continuously releases datasets, including annually updated neurological and psychopathological phenotype data, these can be used to regularly update the normative calculator, minimizing Flynn effects (observed increase in population intelligence quotient (IQ) throughout the twentieth century [56]). It is also worth highlighting that several components of the Enroll-HD cognitive test battery differ from the original test version and mode of application described in the respective foundational publications [16, 17], e.g., Stroop uses 45 s limit to obtain the number of correct answers as the raw score, so we provide the first normative data for this particular mode of application.

Limitations of this study are small sample size for some languages. Additional data from studies other than Enroll-HD could help to expand the normative data collection and to increase comparability of cognitive test results across languages.

### Supplementary Information

Below is the link to the electronic supplementary material.

Supplementary file1 (pdf 1243 KB)

## Data Availability

To allow replication of results, anonymized data of the Enroll-HD are available to any interested, qualified researcher working at a recognized research institution upon request through a straightforward online application procedure. More information on the Enroll-HD study is available at https://enroll-hd.org.
