# Supplementary material for: Establishing normative data for the evaluation of cognitive performance in Huntington’s disease considering the impact of gender, age, language, and education
Source: J Neurol. 2023 Jun 22;270(10):4903–13. doi: 10.1007/s00415-023-11823-x (PMC10511566; doi:10.1007/s00415-023-11823-x)
Supplement: Supplementary file 1 — Supplementary file1 (pdf 1243 KB) [file 415_2023_11823_MOESM1_ESM.pdf]

Supplementary Material Table 1

Comparison of models for regression-based z-scores calculation

Included predictors	Model type	Symbol Digit Modalities Test	Category Fluency	Stroop Color Naming	Stroop Word Reading	Stroop Interference	Trail Making Test-A	Trail Making Test-B	Letter Fluency
age + edu + lan + gen	LMR	23,937.19 (112.75)	19,964.73 (91.61)	25,908.32 (105.84)	27,201.27 (110.30)	22,784.53 (136.48)	1,865.05 (134.49)	2,507.29 (89.57)	21,101.89 (76.94)
	GAM	23,862.85 (114.91) *	19,939.03 (92.36)	25,863.26 (107.09) *	27,126.16 (110.42)	22,749.68 (139.96)	1,801.90 (137.89) *	2,403.16 (90.76)	21,077.55 (76.96) *
age + edu + lan	LMR	24,005.04 (111.45)	19,966.89 (91.39)	25,911.38 (105.85)	27,201.41 (110.30)	22,785.86 (136.19)	1,864.91 (134.09)	2,508.28 (89.66)	21,109.60 (76.84)
	GAM	23,926.45 (113.56)	19,938.69 (92.25) *	25,864.98 (107.24)	27,124.15 (110.46) *	22,749.64 (139.86) *	1,802.61 (137.35)	2,402.11 (90.84) *	21,083.56 (76.88)
age + edu + gen	LMR	24,083.49 (108.82)	20,003.53 (90.16)	25,974.03 (105.74)	27,271.01 (106.67)	22,815.86 (135.19)	2,062.25 (125.00)	2,706.08 (88.95)	21,167.00 (76.53)
	GAM	23,983.17 (111.85)	19,974.61 (91.00)	25,924.09 (106.59)	27,201.02 (107.06)	22,782.65 (138.48)	1,964.28 (128.84)	2,559.34 (89.92)	21,130.12 (76.40)
age + lan + gen	LMR	24,245.68 (111.32)	20,138.20 (90.66)	26,025.43 (102.82)	27,412.77 (107.81)	22,939.38 (133.40)	1,957.77 (131.55)	2,703.40 (90.11)	21,337.21 (74.75)
	GAM	24,176.60 (112.48)	20,019.18 (92.00)	25,974.00 (104.41)	27,365.51 (108.42)	22,895.64 (137.06)	1,895.90 (135.30)	2,605.75 (91.50)	21,314.24 (75.21)
edu + lan + gen	LMR	24,697.14 (101.58)	20,019.18 (92.00)	26,100.68 (103.18)	27,352.21 (107.55)	23,244.06 (118.61)	2,277.46 (120.43)	2,879.00 (88.44)	21,099.87 (76.85)
	GAM	24,618.33 (102.54)	20,011.37 (92.09)	26,075.64 (103.24)	27,279.93 (107.01)	23,213.89 (120.26)	2,227.68 (120.58)	2,810.77 (89.22)	21,084.19 (76.72)

Note: Values represent leave-one-out information criterion (LOO-IC), and its standard deviation (in brackets), smaller values of LOO-IC indicate better fit; *models with the best fit according to LOO-IC are presented in bold; edu = education; lan = language; gen = gender; LMR = linear multiple regression; GAM = Generalized Additive Model.

Supplementary Material Table 2

Posterior mean values for each cognitive test stratified by age

Age (year)	Symbol Digit Modalities Test	Category Fluency Test	Stroop Color Naming Test	Stroop Word Reading Test	Stroop Interference Test	Trail Making Test-A	Trail Making Test -B	Letter Fluency Test
20	56 [55, 58]	23 [22, 24]	78 [76, 80]	99 [97, 102]	48 [47, 50]	22 [21, 23]	46 [43, 49]	41 [39, 43]
30	55 [54, 56]	23 [22, 23]	78 [77, 80]	99 [97, 100]	47 [46, 48]	22 [22, 23]	47 [45, 49]	42 [40, 43]
40	53 [52, 54]	23 [22, 23]	77 [76, 79]	98 [96, 100]	46 [45, 47]	24 [23, 24]	48 [46, 51]	42 [41, 44]
50	50 [49, 51]	22 [22, 23]	76 [74, 77]	96 [95, 98]	43 [42, 44]	25 [25, 26]	52 [50, 54]	43 [41, 44]
60	46 [45, 47]	22 [21, 22]	72 [71, 74]	93 [91, 95]	39 [38, 41]	29 [28, 30]	59 [57, 62]	42 [41, 43]
70	41 [39, 42]	20 [20, 21]	68 [66, 69]	88 [86, 90]	35 [34, 36]	34 [32, 36]	73 [70, 78]	41 [39, 42]
80	34 [31, 37]	18 [17, 20]	62 [58, 65]	82 [78, 86]	30 [27, 32]	43 [39, 47]	100 [89, 113]	39 [36, 42]
90	27 [21, 32]	16 [13, 19]	55 [48, 61]	76 [67, 83]	24 [18, 29]	55 [45, 69]	143 [112, 185]	37 [30, 42]

Note. Values represent posterior means and their 95% Posterior Probability Intervals (PPI, in brackets) for different years of age (rows), grand mean for gender and language and education set on sample median (i.e., 14 years).

Supplementary Material Table 3

Posterior mean values for each cognitive test stratified by years of education

Education (year)	Symbol Digit Modalities Test	Category Fluency Test	Stroop Color Naming Test	Stroop Word Reading Test	Stroop Interference Test	Trail Making Test-A	Trail Making Test -B	Letter Fluency Test
8	42 [40, 43]	20 [19, 21]	70 [68, 72]	86 [84, 89]	38 [37, 40]	30 [28, 32]	69 [65, 74]	34 [32, 36]
10	46 [44, 47]	21 [20, 21]	72 [71, 74]	91 [89, 93]	40 [39, 41]	27 [26, 29]	61 [58, 64]	37 [36, 39]
12	48 [47, 49]	22 [21, 22]	74 [73, 76]	94 [93, 96]	42 [41, 43]	26 [25, 27]	55 [53, 58]	40 [39, 41]
14	50 [49, 51]	23 [22, 23]	76 [75, 77]	97 [95, 98]	44 [43, 45]	25 [24, 26]	51 [49, 53]	43 [41, 44]
16	52 [51, 53]	23 [23, 24]	78 [76, 79]	100 [98, 101]	45 [44, 46]	24 [23, 25]	48 [46, 50]	45 [44, 46]
18	53 [52, 54]	24 [23, 25]	79 [77, 80]	102 [100, 104]	46 [45, 48]	23 [22, 24]	46 [44, 48]	47 [45, 48]
20	55 [53, 56]	25 [24, 25]	80 [78, 81]	103 [101, 105]	47 [46, 49]	23 [22, 24]	45 [43, 47]	48 [46, 49]
22	56 [54, 57]	25 [24, 26]	81 [79, 83]	103 [100, 106]	48 [46, 49]	23 [22, 25]	45 [42, 48]	49 [47, 51]

Note. Values represent posterior means and their 95% Posterior Probability Intervals (PPI, in brackets) for different years of education (rows), grand mean for gender and language and age set on sample median (i.e, 48 years);

Supplementary Material Table 4

Means and standard deviations for each cognitive test for the whole sample (N = 3,267) stratified by age, education, and gender

	Age Groups																	
	18-34 years			35-44 years			45-54 years			55-64 years			> 64 years			Total		
	female	male	all	female	male	all	female	male	all	female	male	all	female	male	all	female	male	all
**Symbol Digit Modalities Test**																		
≤ 12 education years	52.94	50.32	51.73	50.97	48.27	49.63	47.85	43.37	46.07	43.81	40.98	42.96	34.94	34.82	34.89	45.77	43.72	44.93
	(11.36)	(9.58)	(10.62)	(12.24)	(12.01)	(12.15)	(10.93)	(9.61)	(10.63)	(11.51)	(11.48)	(11.55)	(11.95)	(9.79)	(11.02)	(12.89)	(11.78)	(12.49)
> 12 education years	58.52	56.00	57.59	56.69	53.37	55.41	53.2	50.76	52.29	50.27	47.25	49.12	45.41	42.67	44.05	54.07	50.79	52.79
	(9.76)	(10.69)	(10.17)	(9.66)	(9.11)	(9.58)	(10.17)	(9.15)	(9.86)	(10.27)	(9.29)	(10.00)	(9.19)	(9.28)	(9.32)	(10.68)	(10.55)	(10.75)
total	57.52	54.63	56.40	55.66	52.06	54.18	51.86	48.78	50.69	48.1	45.62	47.21	41.32	40.03	40.70	51.96	48.89	50.75
	(10.27)	(10.7)	(10.53)	(10.4)	(10.16)	(10.44)	(10.61)	(9.82)	(10.42)	(11.12)	(10.26)	(10.88)	(11.53)	(10.14)	(10.89)	(11.85)	(11.33)	(11.74)
**Category Fluency Test (Animals)**																		
≤ 12 education years	20.67	21.09	20.87	21.55	19.74	20.65	20.52	21.00	20.71	20.58	21.12	20.74	18.04	19.31	18.60	20.28	20.48	20.36
	(4.95)	(6.28)	(5.6)	(5.35)	(5.18)	(5.32)	(4.90)	(5.93)	(5.33)	(5.42)	(5.27)	(5.37)	(5.46)	(5.40)	(5.45)	(5.31)	(5.67)	(5.46)
> 12 education years	23.56	23.26	23.45	23.48	22.84	23.23	23.19	22.94	23.09	22.57	22.47	22.53	21.39	20.57	20.98	23.06	22.58	22.87
	(5.15)	(5.44)	(5.25)	(5.34)	(5.45)	(5.39)	(5.31)	(5.38)	(5.33)	(5.77)	(5.05)	(5.5)	(4.88)	(5.37)	(5.13)	(5.36)	(5.40)	(5.38)
total	23.05	22.73	22.93	23.13	22.04	22.68	22.52	22.41	22.48	21.89	22.12	21.97	20.07	20.14	20.10	22.35	22.01	22.22
	(5.22)	(5.72)	(5.42)	(5.39)	(5.54)	(5.47)	(5.33)	(5.59)	(5.43)	(5.72)	(5.14)	(5.52)	(5.36)	(5.40)	(5.37)	(5.48)	(5.55)	(5.51)
**Stroop Color Naming Test**																		
≤ 12 education years	73.21	79.51	76.14	73.57	71.94	72.76	73.43	70.74	72.35	70.18	67.45	69.38	62.12	63.9	62.9	70.52	70.93	70.69
	(13.19)	(14.45)	(14.11)	(13.91)	(13.75)	(13.8)	(13.57)	(14.36)	(13.91)	(12.44)	(13.81)	(12.89)	(13.96)	(11.38)	(12.88)	(13.85)	(14.54)	(14.13)
> 12 education years	79.03	79.92	79.35	79.99	77.58	79.06	76.72	76.77	76.74	78.01	73.96	76.46	70.42	67.92	69.19	77.71	75.94	77.03
	(12.34)	(13.04)	(12.6)	(13.7)	(14.69)	(14.13)	(13.34)	(14.26)	(13.67)	(13.77)	(13.93)	(13.96)	(11.23)	(13.06)	(12.21)	(13.3)	(14.31)	(13.72)
total	78.00	79.82	78.70	78.83	76.13	77.72	75.9	75.12	75.61	75.33	72.29	74.25	67.15	66.54	66.86	75.88	74.58	75.37
	(12.68)	(13.38)	(12.98)	(13.94)	(14.64)	(14.28)	(13.46)	(14.51)	(13.86)	(13.83)	(14.16)	(14.01)	(13.00)	(12.62)	(12.81)	(13.8)	(14.54)	(14.11)
**Stroop Word Reading Test**																		
≤ 12 education years	94.31	99.66	96.8	93.13	92.42	92.78	92.81	89.61	91.53	90.43	87.59	89.59	81.04	81.82	81.38	90.4	90.42	90.40
	(15.44)	(19.36)	(17.52)	(16.87)	(15.94)	(16.36)	(15.63)	(16.11)	(15.86)	(17.86)	(17.00)	(17.62)	(17.84)	(13.86)	(16.16)	(17.35)	(17.53)	(17.42)
> 12 education years	99.65	102.35	100.64	101.86	100.18	101.21	99.38	98.85	99.18	97.57	95.18	96.66	91.34	89.25	90.31	98.89	97.96	98.53
	(15.6)	(14.51)	(15.25)	(16.72)	(17.43)	(17.00)	(16.59)	(16.6)	(16.58)	(16.3)	(17.05)	(16.62)	(13.00)	(17.33)	(15.30)	(16.21)	(16.98)	(16.52)
total	98.71	101.69	99.86	100.28	98.19	99.42	97.74	96.34	97.21	95.13	93.21	94.45	87.28	86.70	87.00	96.72	95.92	96.41
	(15.69)	(15.84)	(15.80)	(17.06)	(17.36)	(17.2)	(16.58)	(16.95)	(16.72)	(17.17)	(17.33)	(17.24)	(15.87)	(16.57)	(16.19)	(16.92)	(17.45)	(17.13)

**Stroop Interference Test**																		
≤ 12 education years	44.13 (10.15)	46.45 (9.48)	45.22 (9.88)	42.25 (12.04)	41.12 (9.69)	41.67 (10.87)	40.81 (7.74)	38.82 (9.23)	40.01 (8.4)	38.54 (9.72)	38.03 (10.21)	38.39 (9.85)	32.08 (11.53)	29.68 (9.20)	31.04 (10.62)	39.44 (10.73)	39.17 (10.94)	39.33 (10.81)
> 12 education years	48.48 (10.55)	49.57 (10.87)	48.88 (10.67)	46.70 (8.94)	46.25 (11.19)	46.53 (9.86)	44.13 (10.06)	44.00 (9.84)	44.08 (9.97)	42.73 (9.66)	40.95 (12.22)	42.05 (10.74)	36.80 (8.99)	35.53 (11.05)	36.18 (10.05)	44.91 (10.34)	44.12 (11.88)	44.60 (10.97)
total	47.69 (10.6)	48.78 (10.61)	48.12 (10.61)	45.91 (9.69)	44.90 (11.03)	45.49 (10.27)	43.33 (9.65)	42.62 (9.93)	43.06 (9.76)	41.33 (9.87)	40.20 (11.78)	40.93 (10.60)	34.96 (10.29)	33.55 (10.8)	34.3 (10.54)	43.53 (10.71)	42.77 (11.83)	43.23 (11.17)
**Trail Making Test – Part A**																		
≤ 12 education years	26.66 (9.28)	24.67 (7.94)	25.73 (8.71)	25.32 (8.91)	26.45 (11.23)	25.9 (10.14)	29.61 (11.77)	34.51 (37.98)	31.59 (25.79)	32.21 (12.43)	36.93 (32.92)	33.71 (21.19)	44.91 (25.42)	37.86 (14.79)	41.87 (21.71)	31.92 (15.92)	31.83 (24.72)	31.88 (20.05)
> 12 education years	22.89 (8.69)	22.18 (12.39)	22.62 (10.23)	25.08 (21.12)	23.10 (7.82)	24.32 (17.28)	25.49 (9.43)	24.51 (10.64)	25.13 (9.89)	27.35 (9.82)	26.70 (7.93)	27.11 (9.16)	30.82 (9.88)	34.41 (17.32)	32.52 (13.98)	25.53 (12.77)	25.28 (11.87)	25.44 (12.43)
total	23.57 (8.91)	22.79 (11.5)	23.26 (10.01)	25.12 (19.42)	24.03 (9.00)	24.67 (15.96)	26.51 (10.2)	27.3 (22.35)	26.81 (15.87)	28.94 (10.97)	29.49 (18.93)	29.13 (14.31)	36.51 (19.11)	35.66 (16.48)	36.12 (17.93)	27.16 (13.92)	27.12 (16.77)	27.14 (15.10)
**Trail Making Test – Part B**																		
≤ 12 education years	57.43 (31.54)	60.97 (33.44)	59.09 (32.38)	51.89 (21.43)	59.38 (22.86)	55.74 (22.40)	72.66 (41.62)	68.6 (43.68)	71.02 (42.37)	70.18 (36.88)	78.58 (49.15)	72.85 (41.23)	111.47 (63.2)	93.34 (47.67)	103.66 (57.53)	73.2 (45.43)	71.52 (41.88)	72.50 (43.96)
> 12 education years	45.53 (19.15)	47.33 (22.7)	46.21 (20.55)	47.82 (24.59)	50.81 (22.59)	48.98 (23.85)	51.46 (22.67)	49.61 (21.00)	50.78 (22.07)	54.64 (22.71)	56.81 (27.76)	55.45 (24.71)	70.39 (36.1)	72.97 (38.33)	71.61 (37.10)	51.41 (24.58)	53.57 (26.97)	52.24 (25.54)
total	47.69 (22.35)	50.68 (26.35)	48.87 (24.03)	48.58 (24.05)	53.19 (22.94)	50.50 (23.68)	56.72 (29.94)	54.93 (30.30)	56.05 (30.06)	59.67 (28.97)	62.72 (36.08)	60.76 (31.70)	87.01 (52.76)	80.42 (42.98)	84.01 (48.59)	56.96 (32.64)	58.62 (32.87)	57.61 (32.73)
**Letter Fluency Test Total (the sum of all three letters)**																		
≤ 12 education years	38.01 (10.3)	33.78 (11.71)	36.06 (11.13)	37.22 (10.23)	35.2 (11.89)	36.17 (11.12)	37.28 (11.1)	36.92 (12.04)	37.14 (11.45)	37.88 (10.79)	38.35 (13.77)	38.02 (11.75)	32.41 (12.27)	34.55 (13.47)	33.32 (12.79)	36.73 (11.11)	35.72 (12.57)	36.31 (11.74)
> 12 education years	43.25 (11.25)	42.89 (11.76)	43.12 (11.43)	44.92 (12.36)	42.93 (10.45)	44.15 (11.69)	43.33 (12.69)	43.15 (11.75)	43.26 (12.34)	45.52 (11.86)	42.26 (12.66)	44.29 (12.25)	43.97 (11.87)	40.64 (14.74)	42.42 (13.36)	44.12 (12.01)	42.56 (12.09)	43.52 (12.06)
total	42.3 (11.25)	40.68 (12.36)	41.67 (11.72)	43.5 (12.35)	40.76 (11.39)	42.36 (12.02)	41.83 (12.58)	41.44 (12.13)	41.68 (12.4)	42.98 (12.05)	41.2 (13.05)	42.35 (12.43)	39.27 (13.28)	38.39 (14.54)	38.87 (13.85)	42.23 (12.22)	40.64 (12.6)	41.61 (12.39)
**Letter Fluency Test - 1. Letter**																		
≤ 12 education years	13.37 (4.22)	11.48 (4.15)	12.50 (4.28)	13.64 (4.07)	11.90 (4.66)	12.73 (4.46)	12.81 (4.37)	12.66 (4.36)	12.75 (4.35)	12.89 (4.53)	13.27 (5.26)	13.01 (4.76)	11.23 (4.87)	12.00 (4.82)	11.56 (4.84)	12.77 (4.49)	12.24 (4.65)	12.55 (4.56)
> 12 education years	14.96 (4.56)	14.80 (4.65)	14.90 (4.59)	15.33 (4.89)	15.07 (4.43)	15.23 (4.71)	14.86 (4.85)	14.97 (4.76)	14.9 (4.81)	15.49 (4.86)	14.34 (4.84)	15.06 (4.88)	15.14 (5.03)	14.17 (6.02)	14.69 (5.52)	15.13 (4.80)	14.73 (4.85)	14.98 (4.82)
total	14.67 (4.54)	14.00 (4.74)	14.41 (4.63)	15.02 (4.79)	14.19 (4.71)	14.67 (4.77)	14.36 (4.81)	14.34 (4.75)	14.35 (4.79)	14.63 (4.90)	14.05 (4.97)	14.42 (4.93)	13.55 (5.31)	13.37 (5.69)	13.47 (5.48)	14.53 (4.83)	14.03 (4.92)	14.33 (4.87)
**Letter Fluency Test - 2. Letter**																		

≤ 12 education years	11.58 (3.67)	10.35 (4.25)	11.01 (3.98)	11.24 (4.43)	10.68 (4.44)	10.95 (4.43)	11.66 (4.20)	11.35 (4.43)	11.54 (4.28)	11.33 (4.08)	11.51 (4.73)	11.38 (4.28)	9.99 (4.45)	10.18 (5.23)	10.07 (4.78)	11.20 (4.17)	10.82 (4.61)	11.04 (4.36)
> 12 education years	13.06 (4.18)	13.06 (4.32)	13.06 (4.23)	13.55 (4.86)	12.99 (4.52)	13.34 (4.73)	13.32 (4.52)	13.26 (4.51)	13.3 (4.51)	13.97 (4.38)	12.81 (4.39)	13.53 (4.41)	13.22 (4.06)	12.54 (5.00)	12.91 (4.52)	13.41 (4.43)	12.98 (4.49)	13.25 (4.46)
total	12.79 (4.13)	12.41 (4.45)	12.64 (4.26)	13.13 (4.86)	12.35 (4.61)	12.8 (4.77)	12.91 (4.49)	12.74 (4.56)	12.84 (4.51)	13.09 (4.45)	12.46 (4.51)	12.87 (4.48)	11.91 (4.50)	11.67 (5.19)	11.8 (4.82)	12.85 (4.47)	12.38 (4.63)	12.66 (4.54)
**Letter Fluency Test - 3. Letter**																		
≤ 12 education years	13.07 (5.08)	11.95 (5.00)	12.55 (5.06)	12.35 (4.07)	12.62 (4.78)	12.49 (4.44)	12.81 (4.55)	12.91 (5.11)	12.85 (4.77)	13.66 (4.38)	13.56 (5.82)	13.63 (4.85)	11.19 (4.67)	12.36 (5.49)	11.69 (5.05)	12.76 (4.62)	12.66 (5.23)	12.72 (4.88)
> 12 education years	15.24 (4.74)	15.02 (5.06)	15.16 (4.86)	16.00 (4.95)	14.81 (4.03)	15.54 (4.65)	15.15 (5.02)	14.91 (4.74)	15.06 (4.92)	16.05 (4.78)	15.11 (5.08)	15.70 (4.91)	15.61 (4.53)	13.93 (5.50)	14.83 (5.06)	15.57 (4.85)	14.84 (4.86)	15.29 (4.86)
total	14.84 (4.87)	14.28 (5.20)	14.62 (5.01)	15.33 (5.00)	14.19 (4.36)	14.86 (4.77)	14.57 (5.01)	14.36 (4.92)	14.49 (4.97)	15.26 (4.78)	14.70 (5.32)	15.06 (4.98)	13.81 (5.06)	13.35 (5.53)	13.60 (5.28)	14.85 (4.94)	14.23 (5.06)	14.61 (5.00)

Note. Symbol Digit Modalities Test (min.-max.: 0–110 points in 90 sec); Category fluency (number of correct words - animals in 60 sec); Stroop in each test (min.-max.: 0–200 in 45 sec); Trail Making Test, Part A and Part B (Both time in sec, max. 240 sec, the longer time elapsed, the worse performance); Letter fluency test (number of correct words for three letters in 60 sec each, i.e. in total 180 sec).

Supplementary Material Table 5

Means and standard deviations for each cognitive test for the English sample stratified by age, education, and gender

	Age Group																	
	18-34 years			35-44 years			45-54 years			55-64 years			> 64 years			Total		
	female	male	all	female	male	all	female	male	all	female	male	all	female	male	all	female	male	all
**Symbol Digit Modalities Test**																		
≤ 12 education years	58.09 (9.6)	51.06 (9.08)	54.79 (9.93)	51.11 (14.27)	50.77 (13.93)	50.93 (13.97)	52.33 (8.03)	45.52 (8.19)	49.45 (8.73)	48.55 (10.12)	43.74 (12.6)	46.87 (11.23)	41.47 (9.42)	38.91 (8.91)	40.23 (9.2)	50.17 (11.26)	45.86 (11.56)	48.29 (11.57)
> 12 education years	58.95 (9.55)	57.96 (10.69)	58.59 (9.96)	58.11 (9.43)	54.41 (8.14)	56.85 (9.17)	54.71 (9.36)	52.63 (8.34)	53.92 (9.02)	51.55 (9.66)	48.36 (9.58)	50.46 (9.73)	46.87 (8.98)	43.62 (8.51)	45.33 (8.89)	54.72 (10.25)	51.63 (10.4)	53.57 (10.41)
total	58.83 (9.54)	56.55 (10.72)	57.98 (10.05)	57.14 (10.48)	53.5 (9.97)	55.78 (10.43)	54.25 (9.15)	51.05 (8.8)	53.0 (9.13)	50.85 (9.83)	47.25 (10.52)	49.62 (10.21)	45.56 (9.35)	42.45 (8.82)	44.07 (9.21)	53.87 (10.59)	50.29 (10.94)	52.49 (10.87)
**Categorical Fluency Test (Animals)**																		
≤ 12 education years	21.11 (4.84)	23.35 (6.13)	22.17 (5.56)	21.18 (6.49)	20.03 (5.24)	20.59 (5.85)	21.33 (4.31)	21.21 (4.99)	21.28 (4.58)	20.24 (5.11)	20.94 (5.26)	20.49 (5.15)	18.74 (4.72)	20.22 (5.75)	19.45 (5.25)	20.5 (5.08)	21.15 (5.54)	20.79 (5.29)
> 12 education years	23.67 (5.35)	23.74 (5.02)	23.7 (5.23)	23.25 (5.61)	22.82 (5.4)	23.1 (5.53)	22.45 (4.7)	22.96 (5.28)	22.64 (4.93)	22.07 (5.63)	22.54 (4.69)	22.23 (5.32)	21.33 (4.64)	20.79 (5.52)	21.07 (5.07)	22.68 (5.31)	22.64 (5.24)	22.66 (5.28)
total	23.32 (5.35)	23.66 (5.25)	23.45 (5.31)	22.96 (5.77)	22.12 (5.47)	22.65 (5.67)	22.23 (4.64)	22.57 (5.25)	22.36 (4.88)	21.63 (5.56)	22.16 (4.86)	21.82 (5.33)	20.7 (4.77)	20.65 (5.56)	20.68 (5.15)	22.27 (5.34)	22.29 (5.34)	22.28 (5.34)
**Stroop Color Naming Test**																		
≤ 12 education years	74.32 (15.07)	82.61 (17.75)	78.28 (16.8)	74.93 (13.09)	71.9 (14.91)	73.36 (14.02)	76.29 (12.02)	71.03 (12.08)	74.06 (12.25)	71.95 (12.99)	69.33 (14.76)	71.08 (13.59)	66.09 (15.33)	65.31 (9.98)	65.71 (12.92)	72.72 (13.85)	71.95 (15.06)	72.39 (14.37)
> 12 education years	79.82 (11.57)	82.32 (14.17)	80.7 (12.58)	82.28 (14.79)	78.42 (17.29)	80.97 (15.76)	77.34 (14.01)	78.85 (14.29)	77.92 (14.11)	79.12 (14.38)	75.25 (14.21)	77.79 (14.42)	71.21 (11.07)	69.41 (12.13)	70.36 (11.59)	78.6 (13.72)	77.1 (15.03)	78.04 (14.23)
total	79.08 (12.21)	82.38 (14.92)	80.31 (13.36)	81.26 (14.76)	76.79 (16.91)	79.6 (15.72)	77.14 (13.63)	77.1 (14.17)	77.12 (13.82)	77.41 (14.37)	73.88 (14.5)	76.2 (14.49)	69.96 (12.38)	68.38 (11.73)	69.21 (12.08)	77.5 (13.93)	75.91 (15.18)	76.89 (14.44)
**Stroop Word Reading Test**																		
≤ 12 education years	94.18 (15.98)	101.42 (23.68)	97.63 (20.19)	89.36 (17.14)	86.97 (15.86)	88.12 (16.39)	93.22 (15.78)	84.39 (14.71)	89.49 (15.86)	90.85 (17.43)	86.53 (17.72)	89.38 (17.56)	85.44 (13.67)	82.25 (12.73)	83.89 (13.22)	90.82 (16.33)	88.2 (18.36)	89.68 (17.27)
> 12 education years	98.4 (15.62)	102.75 (13.4)	99.93 (15)	101.03 (18.08)	98.31 (17.42)	100.11 (17.87)	98.4 (16.97)	99.01 (17.47)	98.63 (17.14)	97.81 (16.75)	94.15 (16.5)	96.55 (16.73)	90.93 (13.29)	89.18 (16.33)	90.11 (14.78)	97.89 (16.63)	96.97 (16.79)	97.55 (16.69)
total	97.83 (15.71)	102.47 (15.98)	99.56 (15.95)	99.42 (18.36)	95.47 (17.68)	97.95 (18.18)	97.39 (16.84)	95.75 (17.92)	96.75 (17.26)	96.14 (17.14)	92.35 (17.05)	94.85 (17.18)	89.6 (13.54)	87.42 (15.74)	88.57 (14.63)	96.56 (16.79)	94.93 (17.55)	95.94 (17.1)

**Stroop Interference Test**																		
≤ 12 education years	45.31 (7.41)	46.81 (9.38)	46.05 (8.4)	40.48 (16.35)	41.11 (8.69)	40.82 (12.59)	42.41 (6.16)	37.45 (6.47)	40.21 (6.73)	39.05 (8.16)	38.84 (11.25)	38.98 (9.28)	34.47 (8.88)	33.14 (9.94)	33.81 (9.36)	40.28 (9.73)	39.52 (10.22)	39.93 (9.94)
> 12 education years	49.3 (10.43)	50.79 (10.7)	49.82 (10.54)	48.47 (9.34)	46.21 (10.55)	47.7 (9.81)	44.09 (9.47)	45.51 (9.84)	44.63 (9.62)	43.33 (9.83)	41.14 (13.83)	42.58 (11.38)	37.71 (8.98)	36.02 (10.15)	36.93 (9.55)	45.25 (10.39)	44.25 (12.16)	44.88 (11.08)
total	48.75 (10.16)	49.91 (10.52)	49.19 (10.3)	47.44 (10.78)	44.9 (10.31)	46.48 (10.66)	43.78 (8.97)	43.7 (9.77)	43.75 (9.28)	42.33 (9.62)	40.59 (13.26)	41.73 (11.02)	36.95 (9.03)	35.28 (10.13)	36.17 (9.58)	44.34 (10.44)	43.13 (11.89)	43.88 (11.04)
**Trail Making Test - Part A**																		
≤ 12 education years	24.36 (8.91)	21.52 (6.82)	23.03 (8.06)	23.81 (7.97)	24.76 (8.27)	24.33 (8.06)	24.25 (8.17)	35.15 (41.67)	28.92 (28.2)	28.42 (9.98)	37.87 (43.66)	31.9 (27.8)	33.89 (10.74)	32.0 (9.87)	32.98 (10.28)	27.09 (9.88)	30.42 (28.74)	28.58 (20.59)
> 12 education years	22.29 (9.49)	20.67 (14.63)	21.71 (11.61)	25.47 (27.37)	22.31 (7.39)	24.39 (22.62)	24.18 (10.05)	22.43 (6.46)	23.55 (8.94)	26.2 (8.4)	26.11 (7.04)	26.17 (7.96)	29.74 (9.77)	30.26 (8.71)	29.97 (9.28)	25.08 (14.52)	24.06 (10.15)	24.7 (13.11)
total	22.61 (9.41)	20.86 (13.31)	21.94 (11.07)	25.25 (25.62)	22.95 (7.66)	24.38 (20.73)	24.19 (9.71)	25.32 (21.05)	24.62 (15.0)	26.69 (8.8)	29.05 (22.98)	27.5 (15.22)	30.77 (10.13)	30.73 (9.02)	30.75 (9.61)	25.45 (13.8)	25.6 (16.88)	25.51 (15.04)
**Trail Making Test - Part B**																		
≤ 12 education years	47.94 (16.72)	51.93 (18.57)	49.81 (17.58)	46.9 (16.58)	58.28 (29.19)	53.09 (24.68)	54.81 (23.36)	68.78 (45.99)	60.79 (35.26)	57.7 (25.5)	63.84 (39.24)	59.96 (31.19)	73.0 (32.44)	72.46 (24.31)	72.74 (28.55)	56.39 (25.2)	62.92 (33.46)	59.3 (29.31)
> 12 education years	42.48 (14.27)	44.17 (22.41)	43.08 (17.61)	48.24 (29.52)	44.5 (16.32)	46.94 (25.73)	48.25 (17.14)	45.07 (16.84)	47.1 (17.07)	52.87 (22.16)	57.38 (27.98)	54.36 (24.29)	67.4 (33.58)	69.78 (29.19)	68.47 (31.61)	50.15 (23.81)	51.41 (24.9)	50.61 (24.21)
total	43.31 (14.75)	45.87 (21.8)	44.28 (17.76)	48.06 (28.09)	48.05 (21.13)	48.06 (25.6)	49.45 (18.54)	50.49 (28.08)	49.84 (22.55)	53.93 (22.97)	58.99 (31.13)	55.66 (26.11)	68.79 (33.25)	70.52 (27.84)	69.58 (30.83)	51.3 (24.18)	54.22 (27.65)	52.41 (25.58)
**Letter Fluency Test Total (the sum of all three letters)**																		
≤ 12 education years	39.72 (10.37)	38.14 (12.62)	38.98 (11.4)	38.7 (11.9)	34.96 (13.55)	36.62 (12.84)	37.8 (11.08)	37.57 (9.72)	37.7 (10.42)	40.31 (11.19)	42.45 (13.43)	41.06 (11.98)	38.32 (12.34)	39.46 (15.21)	38.87 (13.67)	39.16 (11.21)	38.61 (13.03)	38.91 (12.04)
> 12 education years	43.69 (11.7)	45.05 (12.36)	44.18 (11.94)	44.69 (11.99)	45.35 (9.71)	44.92 (11.22)	44.73 (12.67)	45.63 (9.78)	45.07 (11.67)	46.39 (11.77)	44.48 (13.75)	45.76 (12.46)	44.84 (11.31)	43.3 (15.26)	44.16 (13.16)	44.89 (11.94)	44.83 (12.24)	44.87 (12.05)
total	43.09 (11.57)	43.57 (12.69)	43.28 (12)	43.91 (12.11)	42.67 (11.68)	43.43 (11.94)	43.47 (12.66)	43.77 (10.31)	43.58 (11.79)	45 (11.89)	43.98 (13.65)	44.66 (12.5)	43.24 (11.85)	42.23 (15.26)	42.78 (13.46)	43.83 (12.01)	43.32 (12.71)	43.63 (12.28)
**Letter Fluency 1. Letter**																		
≤ 12 education years	12.94 (4.58)	13.18 (3.98)	13.05 (4.28)	13.5 (4.75)	11.2 (5.19)	12.22 (5.08)	12.97 (4.29)	12.5 (4.1)	12.76 (4.18)	14.0 (4.61)	14.79 (4.84)	14.28 (4.68)	13.07 (4.61)	13.5 (5.43)	13.28 (4.98)	13.37 (4.53)	13.08 (4.8)	13.24 (4.64)
> 12 education years	14.73 (4.56)	14.93 (4.68)	14.8 (4.6)	14.88 (4.66)	15.68 (4.3)	15.16 (4.55)	15.29 (4.91)	15.48 (4.36)	15.36 (4.71)	15.55 (4.51)	14.67 (5.32)	15.26 (4.8)	15.38 (4.85)	14.94 (6.22)	15.19 (5.47)	15.15 (4.68)	15.12 (4.96)	15.14 (4.78)
total	14.46 (4.6)	14.56 (4.58)	14.5 (4.59)	14.7 (4.68)	14.53 (4.93)	14.63 (4.77)	14.86 (4.88)	14.79 (4.47)	14.84 (4.72)	15.2 (4.57)	14.7 (5.19)	15.03 (4.78)	14.82 (4.87)	14.54 (6.01)	14.69 (5.4)	14.82 (4.7)	14.63 (4.99)	14.75 (4.81)
**Letter Fluency 2. Letter**																		

≤ 12 education years	11.59 (3.26)	10.46 (4.81)	11.07 (4.06)	11.05 (4.52)	10.08 (4.79)	10.51 (4.64)	10.46 (3.28)	10.96 (3.66)	10.68 (3.44)	11.43 (4.12)	11.31 (4.46)	11.39 (4.22)	11.25 (5.06)	10.92 (6.27)	11.09 (5.62)	11.18 (4.01)	10.76 (4.79)	11 (4.38)
> 12 education years	12.44 (4.16)	13.13 (4.4)	12.69 (4.25)	12.61 (4.54)	13 (4.43)	12.75 (4.5)	13.11 (4.53)	13.48 (3.57)	13.25 (4.2)	14.08 (4.38)	13.18 (4.6)	13.78 (4.46)	13.23 (4.03)	13.31 (5.1)	13.27 (4.51)	13.11 (4.38)	13.22 (4.38)	13.15 (4.38)
total	12.31 (4.04)	12.56 (4.6)	12.41 (4.26)	12.41 (4.56)	12.25 (4.68)	12.34 (4.59)	12.63 (4.45)	12.9 (3.73)	12.73 (4.18)	13.48 (4.45)	12.72 (4.62)	13.22 (4.52)	12.75 (4.37)	12.65 (5.52)	12.7 (4.91)	12.75 (4.38)	12.63 (4.6)	12.71 (4.47)
**Letter Fluency 3. Letter**																		
≤ 12 education years	15.19 (4.75)	14.5 (5.09)	14.87 (4.88)	14.15 (4.92)	13.68 (5.38)	13.89 (5.13)	14.37 (5.19)	14.11 (3.86)	14.25 (4.61)	14.89 (4.36)	16.34 (5.47)	15.4 (4.8)	14.0 (4.88)	15.04 (5.96)	14.5 (5.4)	14.6 (4.73)	14.76 (5.19)	14.68 (4.93)
> 12 education years	16.52 (4.74)	16.99 (5.1)	16.69 (4.87)	17.13 (4.71)	16.67 (3.6)	16.97 (4.35)	16.33 (4.79)	16.67 (4.15)	16.46 (4.56)	16.75 (4.78)	16.63 (5.25)	16.71 (4.93)	16.22 (4.18)	15.04 (5.59)	15.71 (4.87)	16.61 (4.69)	16.48 (4.82)	16.57 (4.74)
total	16.32 (4.75)	16.46 (5.18)	16.37 (4.91)	16.75 (4.82)	15.9 (4.3)	16.42 (4.64)	15.97 (4.91)	16.07 (4.21)	16.01 (4.64)	16.33 (4.75)	16.56 (5.29)	16.41 (4.93)	15.68 (4.45)	15.04 (5.66)	15.39 (5.03)	16.24 (4.76)	16.07 (4.96)	16.17 (4.84)

Note. Symbol Digit Modalities Test (min.-max.: 0–110 points in 90 sec); Category fluency (number of correct words - animals in 60 sec); Stroop in each test (min.-max.: 0–200 in 45 sec); Trail Making Test, Part A and Part B (both time in sec, max. 240 sec, the longer time elapsed, the worse performance); Letter fluency (number of correct words for three letters in 60 sec each, i.e. in total 180 sec).

Supplementary Material Table 6

Means and standard deviations for each cognitive test for the German sample stratified by age, education, and gender

	Age Group																	
	18-34 years			35-44 years			45-54 years			55-64 years			> 64 years			Total		
	female	male	all	female	male	all	female	male	all	female	male	all	female	male	all	female	male	all
**Symbol Digit Modalities Test**																		
≤ 12 education years	50.67 (11.25)	55.0 (7.69)	52.17 (10.19)	52.64 (7.85)	47.11 (8.08)	50.48 (8.23)	47.4 (8.49)	43.4 (11.18)	45.4 (9.96)	44.03 (8.85)	35.53 (7.94)	41.43 (9.37)	34.52 (9.52)	33.67 (8.17)	34.13 (8.81)	44.75 (10.92)	40.83 (11.25)	43.2 (11.19)
> 12 education years	58.16 (10.07)	54.57 (9.6)	56.77 (10)	56.45 (9.52)	53.02 (7.1)	54.79 (8.56)	50.96 (9.48)	46.47 (10.01)	49.28 (9.89)	49.2 (10.19)	43.82 (8.77)	46.38 (9.8)	38.43 (8.05)	39.6 (10.91)	39.12 (9.71)	53.07 (10.84)	48.51 (10.43)	51.05 (10.88)
total	56.9 (10.59)	54.64 (9.28)	56.03 (10.14)	55.6 (9.25)	52.05 (7.52)	53.95 (8.64)	50.43 (9.38)	45.77 (10.28)	48.58 (9.98)	46.85 (9.89)	41.75 (9.24)	44.59 (9.9)	36.09 (9.04)	36.79 (10.04)	36.45 (9.51)	50.79 (11.46)	46.69 (11.1)	49.02 (11.48)
**Categorical Fluency Test (Animals)**																		
≤ 12 education years	21.64 (3.93)	22.62 (2.26)	22.0 (3.39)	23.0 (3.44)	18.56 (5.03)	21.26 (4.59)	21.4 (5.84)	20.53 (4.94)	20.97 (5.33)	21.91 (6.09)	23.07 (5.55)	22.27 (5.89)	18.76 (5.16)	21.28 (4.7)	19.92 (5.05)	21.28 (5.35)	21.31 (4.88)	21.29 (5.15)
> 12 education years	24.03 (4.6)	22.21 (5.55)	23.32 (5.05)	23.67 (4.63)	22.65 (5.79)	23.18 (5.22)	25.16 (5.88)	23.08 (5.49)	24.39 (5.81)	25.78 (6.31)	22.31 (5.77)	23.97 (6.24)	20.21 (5.69)	20.45 (4.76)	20.35 (5.08)	24.4 (5.49)	22.37 (5.56)	23.51 (5.61)
total	23.65 (4.56)	22.27 (5.19)	23.12 (4.84)	23.52 (4.38)	21.98 (5.84)	22.81 (5.15)	24.60 (6.00)	22.50 (5.44)	23.77 (5.86)	24.03 (6.46)	22.50 (5.68)	23.35 (6.15)	19.34 (5.34)	20.84 (4.69)	20.12 (5.03)	23.56 (5.62)	22.12 (5.42)	22.94 (5.58)
**Stroop Color Naming Test**																		
≤ 12 education years	74.07 (8.8)	74.62 (11.21)	74.26 (9.45)	72.57 (14.85)	70.67 (15.17)	71.83 (14.66)	71.07 (9.59)	68.6 (10.83)	69.83 (10.13)	70.06 (10.56)	64.2 (12.68)	68.27 (11.45)	62.71 (10.82)	64.17 (10.73)	63.38 (10.66)	69.62 (11.39)	67.38 (12.15)	68.73 (11.71)
> 12 education years	78.66 (12.68)	77.57 (11.0)	78.24 (12.03)	77.82 (13.25)	74.57 (11.12)	76.24 (12.31)	76.0 (11.16)	74.3 (14.59)	75.38 (12.5)	75.46 (11.51)	69.36 (13.04)	72.27 (12.64)	66.5 (13.46)	65.45 (13.64)	65.88 (13.37)	76.5 (12.38)	73.15 (13.1)	75.03 (12.8)
total	77.89 (12.2)	77.13 (10.98)	77.6 (11.72)	76.65 (13.68)	73.93 (11.8)	75.38 (12.85)	75.27 (11.04)	72.98 (13.95)	74.37 (12.27)	73.01 (11.35)	68.07 (13.04)	70.81 (12.33)	64.23 (11.9)	64.84 (12.2)	64.55 (11.98)	74.62 (12.49)	71.78 (13.09)	73.4 (12.82)
**Stroop Word Reading Test**																		
≤ 12 education years	99.6 (12.19)	102.38 (15.55)	100.57 (13.16)	92.79 (15.17)	93.44 (16.69)	93.04 (15.41)	96.47 (12.36)	93.67 (12.83)	95.07 (12.46)	92.53 (20.22)	86.53 (11.08)	90.69 (18.02)	83.43 (15.03)	87.44 (10.37)	85.28 (13.08)	92.3 (16.88)	91.34 (13.4)	91.92 (15.56)
> 12 education years	100.47 (17.89)	103.55 (16.56)	101.67 (17.38)	105.96 (14.26)	101.87 (14.63)	103.98 (14.51)	100.72 (15.15)	99.24 (16.83)	100.17 (15.75)	99.56 (14.83)	94.44 (19.82)	96.88 (17.7)	90.64 (14.15)	90.7 (19.36)	90.68 (17.16)	100.91 (15.94)	98.94 (17.61)	100.04 (16.71)

total	100.33 (17.0)	103.38 (16.29)	101.49 (16.74)	103.03 (15.37)	100.49 (15.15)	101.85 (15.26)	100.09 (14.79)	97.97 (16.09)	99.25 (15.31)	96.37 (17.71)	92.47 (18.27)	94.64 (18.0)	86.31 (14.91)	89.16 (15.64)	87.79 (15.25)	98.56 (16.63)	97.14 (16.99)	97.95 (16.79)
**Stroop Interference Test**																		
≤ 12 education years	44.93 (8.71)	50.5 (6.8)	46.87 (8.38)	44.86 (7.8)	38.44 (10.51)	42.35 (9.3)	38.13 (6.59)	36.47 (6.65)	37.3 (6.56)	36.28 (9.22)	34.73 (8.51)	35.79 (8.94)	33.19 (17.33)	26.72 (8.78)	30.21 (14.25)	38.47 (11.65)	35.37 (10.78)	37.23 (11.38)
> 12 education years	46.91 (9.79)	49.57 (12.5)	47.92 (10.93)	43.88 (7.94)	43.91 (10.23)	43.89 (9.07)	43.78 (10.56)	41.78 (9.53)	43.04 (10.2)	40.12 (10.3)	39.09 (9.66)	39.58 (9.93)	32.36 (9.41)	33.6 (10.69)	33.09 (10.06)	43.5 (10.31)	42.61 (11.45)	43.11 (10.82)
total	46.57 (9.59)	49.7 (11.79)	47.76 (10.55)	44.1 (7.85)	43.02 (10.38)	43.59 (9.1)	42.94 (10.24)	40.55 (9.18)	42.01 (9.88)	38.44 (9.96)	38.0 (9.51)	38.24 (9.73)	32.86 (14.51)	30.34 (10.31)	31.55 (12.48)	42.15 (10.9)	40.88 (11.69)	41.6 (11.26)
**Trail Making Test - Part-A**																		
≤ 12 education years	24.38 (3.8)	28.29 (6.6)	25.75 (5.15)	23.83 (5.95)	26.22 (17.1)	24.86 (11.74)	28.45 (8.44)	27.93 (9.59)	28.15 (8.95)	29.86 (9.14)	34.79 (11.01)	31.5 (9.95)	40.05 (13.23)	36.41 (8.93)	38.33 (11.4)	30.28 (10.76)	31.6 (11.32)	30.84 (10.98)
> 12 education years	21.87 (6.68)	20.51 (8.65)	21.34 (7.5)	21.23 (6.51)	23.03 (6.29)	22.12 (6.42)	25.06 (7.15)	27.32 (17.03)	25.93 (11.93)	28.61 (10.7)	28.71 (9.58)	28.66 (10.04)	35.07 (10.96)	45.0 (30.55)	40.21 (23.41)	24.57 (8.61)	26.53 (15.15)	25.44 (12)
total	22.27 (6.35)	21.6 (8.77)	22.02 (7.35)	21.84 (6.42)	23.64 (9.18)	22.7 (7.88)	25.56 (7.39)	27.49 (15.28)	26.38 (11.4)	29.18 (9.95)	30.35 (10.24)	29.72 (10.06)	37.94 (12.39)	40.44 (21.94)	39.17 (17.65)	26.15 (9.58)	27.86 (14.4)	26.9 (11.96)
**Trail Making Test - Part-B**																		
≤ 12 education years	42.92 (9.16)	47.14 (11.71)	44.4 (10.03)	45.83 (12.04)	61.0 (22.59)	52.33 (18.52)	70.18 (28.91)	61.73 (21.37)	65.31 (24.66)	60.32 (15.78)	87.86 (49.56)	69.5 (33.4)	113.26 (62.92)	101.94 (58.56)	107.92 (60.31)	68.93 (41.84)	76.9 (44.79)	72.34 (43.16)
> 12 education years	43.49 (17.96)	42.81 (18.88)	43.23 (18.24)	42.9 (14.71)	55.03 (21.16)	48.88 (19.07)	49.59 (14.43)	52.8 (18.14)	50.83 (15.95)	56.15 (17.93)	59.84 (32.68)	58.13 (26.74)	83.79 (29.19)	88.07 (65.98)	86.0 (50.76)	49.71 (19.96)	55.4 (31.23)	52.24 (25.71)
total	43.4 (16.8)	43.42 (18.02)	43.41 (17.21)	43.59 (14.07)	56.17 (21.32)	49.62 (18.91)	52.61 (18.55)	55.24 (19.29)	53.72 (18.84)	58.07 (16.97)	67.38 (39.48)	62.35 (29.76)	100.76 (52.84)	95.44 (61.52)	98.14 (56.89)	55.03 (29.02)	61.05 (36.45)	57.68 (32.61)
**Letter Fluency Test Total (the sum of all three letters)**																		
≤ 12 education years	39.0 (9.03)	33.86 (12.56)	37.2 (10.38)	36.55 (7.39)	35.22 (13.05)	35.95 (10.04)	36.33 (10.79)	30.6 (9.97)	33.15 (10.55)	36.11 (6.95)	33.07 (15.25)	35.1 (10.38)	29.32 (7.33)	31.65 (7.45)	30.42 (7.37)	35.1 (8.54)	32.48 (11.28)	33.98 (9.85)
> 12 education years	41.81 (11.06)	40.6 (9.59)	41.34 (10.49)	40.03 (13.23)	39.43 (8.65)	39.73 (11.14)	40.1 (12.4)	36.83 (11.35)	38.79 (12.04)	43.24 (12.53)	37.97 (10.49)	40.46 (11.71)	41.46 (17.08)	32.5 (12.32)	36.81 (15.21)	41.19 (12.42)	38.22 (10.4)	39.87 (11.65)
total	41.36 (10.76)	39.66 (10.19)	40.71 (10.54)	39.24 (12.19)	38.61 (9.64)	38.94 (10.97)	39.49 (12.17)	35.19 (11.26)	37.64 (11.93)	39.97 (10.88)	36.63 (12.02)	38.45 (11.48)	34.25 (13.45)	32.03 (9.78)	33.16 (11.74)	39.49 (11.78)	36.71 (10.92)	38.26 (11.48)
**Letter Fluency Test - 1. Letter**																		
≤ 12 education years	14.38 (4.25)	11.0 (4.47)	13.2 (4.53)	14.73 (4.03)	12.11 (3.48)	13.55 (3.93)	13.67 (3.39)	10.93 (3.53)	12.15 (3.68)	12.39 (3.44)	10.86 (6.69)	11.88 (4.74)	10.32 (3.35)	10.76 (3.44)	10.53 (3.35)	12.72 (3.87)	11.05 (4.39)	12.01 (4.17)
> 12 education years	15.28 (4.82)	15.84 (4.4)	15.5 (4.65)	15.0 (4.88)	14.34 (3.96)	14.67 (4.43)	14.0 (4.56)	13.14 (4.75)	13.66 (4.63)	15.03 (5.55)	13.35 (4.49)	14.14 (5.05)	15.15 (6.82)	10.93 (5.48)	12.96 (6.41)	14.81 (4.99)	13.94 (4.66)	14.42 (4.86)

total	15.14 (4.72)	15.16 (4.68)	15.15 (4.68)	14.94 (4.67)	13.91 (3.94)	14.44 (4.33)	13.95 (4.37)	12.56 (4.54)	13.35 (4.48)	13.82 (4.85)	12.67 (5.23)	13.29 (5.04)	12.28 (5.51)	10.84 (4.4)	11.57 (5.01)	14.23 (4.79)	13.18 (4.76)	13.76 (4.8)
**Letter Fluency Test - 2. Letter**																		
≤ 12 education years	12.08 (3.55)	10.71 (3.59)	11.6 (3.53)	10.36 (2.8)	10.67 (5.07)	10.5 (3.87)	11.5 (5.49)	9.53 (3.96)	10.41 (4.71)	10.64 (2.57)	11.57 (5.37)	10.95 (3.7)	9.53 (3.03)	10.12 (2.71)	9.81 (2.86)	10.7 (3.43)	10.45 (4.11)	10.59 (3.72)
> 12 education years	12.62 (3.51)	11.65 (3.41)	12.24 (3.49)	12.13 (5.18)	11.95 (3.56)	12.04 (4.42)	12.57 (4.42)	11.98 (4.39)	12.33 (4.39)	13.82 (4.8)	12.03 (3.96)	12.87 (4.44)	13.0 (4.71)	11.21 (4.34)	12.07 (4.52)	12.73 (4.37)	11.84 (3.85)	12.33 (4.16)
total	12.53 (3.5)	11.52 (3.41)	12.15 (3.49)	11.73 (4.78)	11.7 (3.87)	11.72 (4.34)	12.4 (4.58)	11.33 (4.38)	11.94 (4.51)	12.36 (4.22)	11.9 (4.34)	12.15 (4.26)	10.94 (4.11)	10.61 (3.52)	10.78 (3.8)	12.16 (4.22)	11.47 (3.96)	11.86 (4.12)
**Letter Fluency Test - 3. Letter**																		
≤ 12 education years	12.54 (4.1)	12.14 (5.67)	12.4 (4.56)	11.45 (2.54)	12.44 (5.05)	11.9 (3.8)	11.17 (3.33)	10.13 (4.05)	10.59 (3.71)	13.07 (3.39)	10.64 (5.11)	12.26 (4.14)	9.47 (2.91)	10.76 (2.93)	10.08 (2.95)	11.67 (3.51)	10.98 (4.33)	11.38 (3.88)
> 12 education years	13.91 (4.2)	13.12 (3.11)	13.6 (3.82)	12.89 (4.69)	12.92 (3.14)	12.91 (3.97)	13.52 (5.0)	11.71 (3.74)	12.8 (4.6)	14.39 (3.95)	12.59 (3.69)	13.44 (3.9)	13.31 (6.26)	10.36 (3.99)	11.78 (5.32)	13.66 (4.62)	12.4 (3.53)	13.1 (4.21)
total	13.69 (4.19)	12.98 (3.51)	13.42 (3.94)	12.57 (4.32)	12.83 (3.53)	12.69 (3.94)	13.15 (4.83)	11.3 (3.85)	12.35 (4.51)	13.79 (3.73)	12.06 (4.17)	13.0 (4.01)	11.03 (4.88)	10.58 (3.39)	10.81 (4.18)	13.1 (4.42)	12.03 (3.8)	12.63 (4.19)

Note: Symbol Digit Modalities Test (min.-max.: 0–110 points in 90 sec); Category fluency (number of correct words - animals in 60 sec); Stroop in each test (min.-max.: 0–200 in 45 sec); Trail Making Test, Part A and Part B (both time in sec, max. 240 sec, the longer time elapsed, the worse performance); Letter fluency test (number of correct words for three letters in 60 sec each, i.e. in total 180 sec).

Supplementary Material Table 7

Means and standard deviations for each cognitive test for the Spanish sample stratified by age, education, and gender.

	Age Group																	
	18-34 years			35-44 years			45-54 years			55-64 years			> 64 years			Total		
	female	male	all	female	male	all	female	male	all	female	male	all	female	male	all	female	male	all
**Symbol Digit Modalities Test**																		
≤ 12 education years	50.38	53.54	51.96	50.9	47.84	48.9	45.96	43.67	45.33	38.46	45.0	39.77	26.35	27.88	26.84	41.22	45.29	42.76
	(13.59)	(7.26)	(10.79)	(14.79)	(11.04)	(12.28)	(12.32)	(9.06)	(11.43)	(12.15)	(10.05)	(11.92)	(10.88)	(9.6)	(10.31)	(14.86)	(12.21)	(14.01)
> 12 education years	61.65	57.14	60.08	54.0	55.53	54.53	55.96	51.0	54.66	42.42	50.2	44.71	38.0	32.67	36.22	54.19	53.02	53.82
	(9.26)	(11.43)	(10.16)	(9.28)	(9.91)	(9.41)	(14.43)	(7.83)	(13.1)	(15.37)	(3.77)	(13.39)	(8.05)	(15.53)	(10.39)	(13.4)	(11.25)	(12.73)
total	57.9	55.41	56.88	53.18	51.24	52.26	51.35	47.53	50.32	39.65	47.17	41.38	29.39	29.18	29.32	47.97	48.82	48.27
	(11.98)	(9.65)	(11.08)	(10.86)	(11.1)	(10.94)	(14.28)	(9.01)	(13.13)	(13.12)	(8.21)	(12.51)	(11.32)	(10.85)	(11.01)	(15.51)	(12.35)	(14.47)
**Categorical Fluency Test (Animals)**																		
≤ 12 education years	18.54	18.62	18.58	22.0	20.26	20.86	20.79	23.6	21.62	20.14	20.71	20.26	17.24	15.88	16.8	19.75	19.91	19.81
	(6.95)	(5.85)	(6.29)	(5.16)	(5.55)	(5.39)	(4.13)	(8.96)	(5.95)	(5.48)	(3.64)	(5.12)	(4.89)	(3.6)	(4.49)	(5.36)	(6.2)	(5.67)
> 12 education years	22.96	23.21	23.05	25.07	23.53	24.52	23.18	25.2	23.71	21.82	21.0	21.56	20.5	19.0	20.0	23.33	23.23	23.3
	(5.09)	(5.21)	(5.06)	(5.49)	(5.71)	(5.55)	(4.85)	(7.22)	(5.54)	(4.21)	(7.55)	(5.21)	(5.24)	(7.94)	(5.79)	(5.12)	(6.17)	(5.46)
total	21.49	21.0	21.29	24.24	21.71	23.03	22.08	24.4	22.72	20.62	20.83	20.67	18.09	16.73	17.65	21.59	21.41	21.53
	(6.06)	(5.9)	(5.96)	(5.51)	(5.77)	(5.74)	(4.64)	(7.96)	(5.79)	(5.15)	(5.29)	(5.13)	(5.08)	(4.88)	(4.98)	(5.52)	(6.38)	(5.83)
**Stroop Color Naming Test**																		
≤ 12 education years	77.85	77.54	77.69	73.5	73.58	73.55	73.12	73.7	73.29	67.82	71.86	68.63	54.53	60.62	56.48	68.78	72.47	70.19
	(13.82)	(10.79)	(12.15)	(17.67)	(13.5)	(14.75)	(12.54)	(11.24)	(12)	(11.3)	(12.29)	(11.44)	(12.7)	(16.52)	(13.98)	(14.81)	(13.45)	(14.37)
> 12 education years	82.62	80.57	81.9	76.11	80.67	77.7	77.24	73.8	76.36	72.45	71.6	72.19	65.5	41.0	59.38	77.09	76.43	76.88
	(9.03)	(12.03)	(10.08)	(12.08)	(11.09)	(11.82)	(13.96)	(18.29)	(15.01)	(12.79)	(9.37)	(11.51)	(7.34)	(7.07)	(13.2)	(12.4)	(15.07)	(13.25)
total	81.03	79.11	80.24	75.42	76.71	76.03	75.38	73.75	74.93	69.13	71.75	69.75	57.39	56.7	57.18	73.11	74.24	73.51
	(10.92)	(11.34)	(11.04)	(13.56)	(12.82)	(13.14)	(13.37)	(14.77)	(13.68)	(11.76)	(10.7)	(11.47)	(12.4)	(16.92)	(13.65)	(14.2)	(14.26)	(14.2)
**Stroop Word Reading Test**																		
≤ 12 education years	103.69	104.08	103.88	101.2	100.37	100.66	96.04	101.5	97.65	90.46	100.71	92.51	74.24	74.75	74.4	91.96	97.86	94.21
	(10.37)	(15.97)	(13.19)	(16.92)	(14.67)	(15.18)	(15.85)	(17.79)	(16.36)	(17.13)	(11.56)	(16.55)	(19.8)	(16.85)	(18.55)	(18.83)	(17.76)	(18.59)
> 12 education years	111.46	111.79	111.58	106.96	113.27	109.16	101.9	100.0	101.41	95.0	105.0	98.12	92.83	86.67	90.78	104.5	107.43	105.44
	(12.59)	(15.65)	(13.54)	(13.07)	(15.8)	(14.22)	(19.28)	(13.72)	(17.87)	(14.45)	(14.98)	(14.9)	(6.55)	(30.66)	(16.48)	(15.8)	(17.32)	(16.3)
total	108.87	108.07	108.55	105.45	106.06	105.74	99.25	100.75	99.66	91.74	102.5	94.27	79.09	78.0	78.74	98.49	102.18	99.79
	(12.33)	(15.99)	(13.83)	(14.18)	(16.29)	(15.11)	(17.89)	(15.48)	(17.17)	(16.36)	(12.62)	(16.11)	(19.1)	(20.44)	(19.23)	(18.38)	(18.12)	(18.35)

**Stroop Interference Test**																		
≤ 12 education years	46.23 (16.66)	47.23 (10.54)	46.73 (13.67)	45.9 (9.07)	44.0 (10.56)	44.66 (9.95)	41.33 (8.55)	43.1 (10.05)	41.85 (8.9)	38.52 (13.7)	40.5 (10.29)	38.88 (13.02)	28.25 (5.74)	24.62 (3.58)	27.04 (5.34)	39.38 (12.66)	41.45 (11.85)	40.17 (12.35)
> 12 education years	51.88 (8.82)	53.36 (12.83)	52.4 (10.26)	46.48 (8.13)	56.47 (13.04)	50.05 (11.11)	48.14 (11.03)	44.6 (11.44)	47.21 (11.1)	38.36 (4.92)	42.4 (9.21)	39.62 (6.52)	31.33 (6.92)	12.0 (4.24)	26.5 (10.81)	46.55 (10.34)	49.48 (15.13)	47.49 (12.1)
total	50.0 (12.09)	50.41 (11.98)	50.17 (11.95)	46.32 (8.27)	49.5 (13.13)	47.85 (10.91)	45.0 (10.45)	43.85 (10.51)	44.68 (10.4)	38.47 (11.77)	41.36 (9.37)	39.12 (11.25)	29.09 (6.08)	22.1 (6.35)	26.91 (6.9)	43.12 (12.02)	45.07 (13.95)	43.8 (12.74)
**Trail Making Test - Part-A**																		
≤ 12 education years	31.55 (10.57)	26.42 (6.99)	28.87 (9.06)	30.1 (12.15)	28.0 (11.78)	28.75 (11.73)	35.62 (12.58)	50.6 (67.07)	40.03 (37.22)	41.74 (16.02)	37.17 (16.7)	40.91 (15.98)	61.31 (40.59)	53.57 (21.92)	58.96 (35.6)	41.03 (23.23)	36.32 (32.29)	39.26 (26.98)
> 12 education years	23.64 (7.21)	22.36 (6.61)	23.18 (6.94)	26.21 (7.76)	21.31 (6.14)	24.49 (7.53)	29.86 (8.37)	28.9 (11.38)	29.61 (9.1)	35.0 (10.47)	27.0 (4.12)	32.5 (9.61)	33.2 (4.76)	47.33 (33.53)	38.5 (19.69)	28.03 (8.74)	25.69 (12.08)	27.27 (9.97)
total	26.06 (9.01)	24.23 (6.96)	25.29 (8.2)	27.35 (9.24)	25.19 (10.25)	26.32 (9.72)	32.52 (10.81)	39.75 (48.13)	34.53 (26.73)	39.79 (14.82)	32.55 (13.21)	38.16 (14.66)	54.62 (37.29)	51.7 (24.06)	53.68 (33.21)	34.35 (18.5)	31.44 (25.56)	33.33 (21.25)
**Trail Making Test - Part-B**																		
≤ 12 education years	83.36 (53.01)	81.42 (55.53)	82.35 (53.1)	53.7 (21.19)	58.33 (13.04)	56.68 (16.18)	98.71 (59.8)	70.9 (62.19)	90.53 (60.93)	107.0 (51.88)	109.33 (77.8)	107.44 (56.11)	152.81 (65.83)	133.0 (60.35)	146.78 (63.52)	104.02 (60.59)	81.57 (55.07)	95.52 (59.38)
> 12 education years	51.08 (27.22)	59.36 (23)	54.05 (25.79)	52.83 (22.67)	46.38 (21.59)	50.57 (22.21)	55.93 (21.25)	54.3 (41.18)	55.5 (27.25)	74.27 (30.09)	50.8 (6.42)	66.94 (27.22)	95.0 (81.71)	111.67 (67.26)	101.25 (71.99)	58.1 (31.15)	57.02 (32.93)	57.75 (31.63)
total	60.94 (39.22)	69.54 (41.92)	64.55 (40.27)	53.09 (21.93)	53.32 (17.85)	53.2 (19.93)	75.67 (48.12)	62.6 (52.03)	72.04 (49.22)	97.27 (48.49)	82.73 (63.07)	93.94 (51.82)	139.05 (72.26)	126.6 (59.49)	135.03 (67.66)	80.29 (52.84)	70.3 (47.64)	76.77 (51.2)
**Letter Fluency Test Total (the sum of all three letters)**																		
≤ 12 education years	35 (12.68)	28.25 (6.63)	31.62 (10.48)	36.8 (10.62)	35.33 (10.99)	35.86 (10.69)	35.48 (9.48)	43.6 (18.8)	37.94 (13.25)	30.35 (9.97)	39.67 (8.36)	32.09 (10.25)	25.75 (12.9)	28.43 (10)	26.57 (11.93)	32.24 (11.34)	34.87 (12.7)	33.24 (11.89)
> 12 education years	41.16 (8.65)	40.31 (10.94)	40.87 (9.36)	46.71 (11.79)	46.46 (11.62)	46.62 (11.56)	43.89 (9.93)	41.9 (17.55)	43.37 (12.15)	39.82 (10.36)	44.2 (12.76)	41.19 (10.93)	38.4 (7.89)	39.67 (11.59)	38.88 (8.63)	43.11 (10.23)	42.89 (12.8)	43.04 (11.08)
total	39.16 (10.37)	34.52 (10.86)	37.29 (10.73)	43.79 (12.19)	40 (12.39)	41.98 (12.34)	40.1 (10.52)	42.75 (17.72)	40.85 (12.87)	33.16 (10.87)	41.73 (10.28)	35.12 (11.23)	28.76 (12.95)	31.8 (11.22)	29.74 (12.32)	37.86 (12.05)	38.51 (13.3)	38.08 (12.48)
**Letter Fluency Test - 1. Letter**																		
≤ 12 education years	11.75 (4.16)	9.25 (3.05)	10.5 (3.79)	12.9 (3.48)	11.94 (4.75)	12.29 (4.29)	11.48 (3.3)	13.5 (5.68)	12.09 (4.18)	9.23 (3.91)	13.33 (2.16)	10.0 (3.97)	8.75 (5.25)	10.71 (3.35)	9.35 (4.76)	10.51 (4.21)	11.62 (4.37)	10.93 (4.29)
> 12 education years	13.92 (3.29)	13.0 (5.18)	13.61 (3.99)	15.62 (4.17)	16.23 (3.96)	15.84 (4.05)	14.54 (3.91)	14.2 (5.65)	14.45 (4.35)	13.91 (4.99)	13.8 (3.11)	13.88 (4.38)	12.2 (4.27)	14.67 (3.51)	13.12 (3.94)	14.45 (3.99)	14.43 (4.66)	14.45 (4.2)
total	13.22 (3.68)	11.2 (4.62)	12.4 (4.17)	14.82 (4.12)	13.74 (4.86)	14.31 (4.49)	13.16 (3.93)	13.85 (5.53)	13.35 (4.4)	10.62 (4.72)	13.55 (2.5)	11.29 (4.46)	9.57 (5.15)	11.9 (3.73)	10.32 (4.81)	12.54 (4.54)	12.9 (4.7)	12.67 (4.59)
**Letter Fluency Test - 2. Letter**																		

≤ 12 education years	10.83 (4.63)	9.42 (3.4)	10.12 (4.04)	11.0 (5.01)	10.78 (4.08)	10.86 (4.34)	11.83 (4.11)	13.7 (6.9)	12.39 (5.07)	9.69 (3.17)	13.33 (4.76)	10.38 (3.72)	8.19 (5.06)	8.29 (4.5)	8.22 (4.8)	10.29 (4.33)	10.98 (4.92)	10.55 (4.56)
> 12 education years	12.48 (3.92)	13.23 (3.83)	12.74 (3.85)	14.42 (3.89)	14.46 (4.77)	14.43 (4.15)	14.29 (3.8)	13.0 (7.48)	13.95 (4.95)	12.27 (4.22)	14.0 (6.2)	12.81 (4.78)	13.4 (4.83)	11.33 (4.93)	12.62 (4.63)	13.55 (3.98)	13.5 (5.25)	13.53 (4.41)
total	11.95 (4.17)	11.4 (4.05)	11.73 (4.1)	13.41 (4.46)	12.32 (4.69)	12.89 (4.57)	13.18 (4.09)	13.35 (7.01)	13.23 (5.03)	10.46 (3.66)	13.64 (5.18)	11.19 (4.22)	9.43 (5.39)	9.2 (4.59)	9.35 (5.07)	11.97 (4.45)	12.12 (5.2)	12.03 (4.72)
**Letter Fluency Test - 3. Letter**																		
≤ 12 education years	12.42 (6.27)	9.58 (2.81)	11.0 (4.97)	12.9 (3.9)	12.61 (3.62)	12.71 (3.65)	12.17 (3.88)	16.4 (7.47)	13.45 (5.47)	11.42 (4.54)	13.0 (3.58)	11.72 (4.37)	8.81 (3.39)	9.43 (4.12)	9.0 (3.54)	11.45 (4.51)	12.26 (4.99)	11.76 (4.69)
> 12 education years	14.76 (3.69)	14.08 (4.37)	14.53 (3.89)	16.67 (4.98)	15.77 (4.02)	16.35 (4.63)	15.07 (3.73)	14.7 (6.34)	14.97 (4.47)	13.64 (4.01)	16.4 (4.1)	14.5 (4.12)	12.8 (3.9)	13.67 (3.51)	13.12 (3.52)	15.11 (4.18)	14.95 (4.61)	15.06 (4.3)
total	14.0 (4.73)	11.92 (4.29)	13.16 (4.63)	15.56 (4.95)	13.94 (4.05)	14.78 (4.58)	13.76 (4.03)	15.55 (6.8)	14.27 (4.98)	12.08 (4.46)	14.55 (4.03)	12.65 (4.45)	9.76 (3.83)	10.7 (4.27)	10.06 (3.93)	13.34 (4.7)	13.48 (4.98)	13.39 (4.79)

Note: Symbol Digit Modalities Test (min.-max.: 0–110 points in 90 sec); Category fluency (number of correct words - animals in 60 sec); Stroop in each test (min.-max.: 0–200 in 45 sec); Trail Making Test, Part A and Part B (both time in sec, max. 240 sec, the longer time elapsed, the worse performance); Letter fluency test (number of correct words for three letters in 60 sec each, i.e. in total 180 sec).

Supplementary Material Table 8

Means and standard deviations for each cognitive test for the Italian sample stratified by age, education, and gender.

	Age Group																	
	18-34 years			35-44 years			45-54 years			55-64 years			> 64 years			Total		
	female	male	all	female	male	all	female	male	all	female	male	all	female	male	all	female	male	all
**Symbol Digit Modalities Test**																		
≤ 12 education years	42.25 (9.18)	46.43 (13.54)	44.2 (11.19)	47.78 (12.04)	41.17 (5.85)	45.13 (10.31)	40.21 (10.13)	32.56 (7.43)	37.22 (9.77)	32.62 (11.13)	37.25 (6.08)	33.71 (10.2)	25.8 (10.89)	26.17 (10.59)	26.0 (10.18)	38.45 (12.27)	36.59 (11.34)	37.72 (11.88)
> 12 education years	54.26 (10.67)	46.52 (7.87)	51.14 (10.3)	51.76 (12.17)	49.35 (10.92)	50.38 (11.38)	44.61 (10.59)	48.58 (10.82)	46.2 (10.68)	39.57 (9.4)	46.75 (11.21)	42.18 (10.45)	NA (NA)	36.75 (8.73)	36.75 (8.73)	49.18 (12.02)	47.27 (10.04)	48.31 (11.16)
total	51.98 (11.35)	46.5 (9.22)	49.69 (10.79)	50.38 (12.04)	47.66 (10.55)	48.95 (11.25)	42.69 (10.46)	41.71 (12.35)	42.3 (11.14)	36.22 (10.67)	43.58 (10.58)	38.49 (11.05)	25.8 (10.89)	30.4 (10.84)	28.87 (10.7)	45.2 (13.14)	43.92 (11.54)	44.64 (12.46)
**Categorical Fluency Test (Animals)**																		
≤ 12 education years	21.88 (3.8)	20.71 (7.59)	21.33 (5.68)	20.22 (5.31)	17.17 (3.6)	19.0 (4.81)	17.0 (3.51)	17.89 (7.77)	17.35 (5.42)	19.92 (6.44)	22.25 (3.3)	20.47 (5.84)	16.83 (7.94)	15.43 (3.69)	16.08 (5.79)	19.1 (5.48)	18.36 (6.07)	18.81 (5.7)
> 12 education years	21.79 (4.73)	22.74 (7.93)	22.18 (6.17)	22.47 (6.08)	22.21 (4.46)	22.32 (5.13)	19.94 (5.26)	20.33 (4.72)	20.1 (4.97)	20.36 (4.8)	21.75 (5.28)	20.86 (4.9)	NA (NA)	15.5 (1.91)	15.5 (1.91)	21.29 (5.15)	21.63 (5.98)	21.45 (5.53)
total	21.81 (4.52)	22.27 (7.77)	22.0 (6.04)	21.69 (5.82)	21.2 (4.72)	21.43 (5.21)	18.66 (4.75)	19.29 (6.16)	18.91 (5.3)	20.15 (5.54)	21.92 (4.56)	20.69 (5.26)	16.83 (7.94)	15.45 (3.05)	15.94 (5.09)	20.47 (5.36)	20.6 (6.17)	20.52 (5.72)
**Stroop Color Naming Test**																		
≤ 12 education years	70.0 (11.67)	83.14 (8.3)	76.13 (11.99)	71.11 (13.23)	67.33 (9.18)	69.6 (11.57)	67.21 (12.9)	61.33 (26.46)	64.91 (19.01)	64.46 (13.09)	59.25 (14.8)	63.24 (13.22)	62.0 (9.19)	58.86 (13.87)	60.31 (11.58)	67.02 (12.33)	66.27 (18.58)	66.72 (15.03)
> 12 education years	72.62 (15.27)	72.65 (12.12)	72.63 (13.97)	73.76 (8.65)	79.79 (11.45)	77.29 (10.7)	72.0 (15.35)	71.83 (10.64)	71.93 (13.46)	72.14 (10.88)	80.38 (14.42)	75.14 (12.61)	NA (NA)	60.5 (23.61)	60.5 (23.61)	72.64 (13.28)	75.11 (13.36)	73.78 (13.33)
total	72.12 (14.56)	75.1 (12.08)	73.36 (13.57)	72.85 (10.27)	77.3 (12.01)	75.23 (11.36)	69.91 (14.31)	67.33 (19.25)	68.89 (16.32)	68.44 (12.39)	73.33 (17.33)	69.95 (14.04)	62.0 (9.19)	59.45 (16.84)	60.35 (14.32)	70.53 (13.17)	72.31 (15.67)	71.31 (14.32)
**Stroop Word Reading Test**																		
≤ 12 education years	81.25 (16.59)	101.43 (2.99)	90.67 (15.81)	95.0 (19.98)	94.83 (13.48)	94.93 (17.12)	86.0 (18.17)	87.78 (11.04)	86.7 (15.5)	81.54 (17.02)	91.0 (24.47)	83.76 (18.62)	74.83 (27.52)	73.86 (20.06)	74.31 (22.74)	84.36 (19.41)	89.39 (16.83)	86.36 (18.49)
> 12 education years	99.09 (15.34)	95.74 (15.02)	97.74 (15.17)	103.0 (15.56)	102.08 (13.08)	102.46 (13.98)	96.22 (14.22)	101.0 (11.1)	98.13 (13.07)	88.5 (15.88)	101.75 (10.55)	93.32 (15.35)	NA (NA)	92.25 (23.27)	92.25 (23.27)	97.48 (15.67)	99.25 (13.85)	98.3 (14.83)
total	95.69 (16.93)	97.07 (13.38)	96.26 (15.46)	100.23 (17.26)	100.63 (13.26)	100.45 (15.1)	91.75 (16.61)	95.33 (12.71)	93.17 (15.16)	85.15 (16.5)	98.17 (16.19)	89.15 (17.3)	74.83 (27.52)	80.55 (22.14)	78.53 (23.47)	92.55 (18.25)	96.12 (15.48)	94.12 (17.15)

**Stroop Interference Test**																		
≤ 12 education years	38.25 (7.87)	46.0 (8.64)	41.87 (8.89)	38.75 (9.13)	34.83 (7.14)	37.07 (8.28)	39.75 (6.48)	36.5 (11.47)	38.67 (8.27)	41.0 (6.4)	43.5 (9.26)	41.77 (7.08)	30.8 (9.65)	26.75 (4.43)	29.0 (7.65)	38.48 (7.89)	38.19 (10.42)	38.36 (8.89)
> 12 education years	45.93 (8.59)	42.83 (7.36)	44.56 (8.14)	43.88 (7.39)	44.71 (12.1)	44.35 (10.21)	39.31 (12.7)	39.33 (9.08)	39.32 (11.1)	42.0 (7.53)	46.86 (9.34)	43.79 (8.34)	NA (NA)	47.5 (19.43)	47.5 (19.43)	43.38 (9.43)	43.49 (10.35)	43.44 (9.85)
total	44.27 (8.93)	43.57 (7.65)	43.96 (8.32)	42.17 (8.19)	42.52 (11.83)	42.35 (10.18)	39.5 (10.33)	38.39 (9.69)	39.07 (9.99)	41.57 (6.92)	45.64 (8.99)	42.97 (7.8)	30.8 (9.65)	37.12 (17.13)	34.69 (14.57)	41.59 (9.18)	41.97 (10.6)	41.76 (9.82)
**Trail Making Test - Part-A**																		
≤ 12 education years	34.17 (12.98)	34.0 (12.23)	34.08 (12.03)	25.38 (8.35)	31.17 (11.6)	27.86 (9.91)	38.18 (14.97)	35.2 (8.93)	37.25 (13.14)	30.83 (7.88)	36.75 (9.22)	33.2 (8.5)	49.25 (8.96)	53.6 (18.69)	51.67 (14.49)	34.57 (13.16)	37.77 (14.18)	35.93 (13.58)
> 12 education years	28.7 (8.59)	28.1 (9.06)	28.45 (8.7)	32.21 (12.51)	25.71 (9.3)	28.65 (11.17)	33.14 (11.02)	31.0 (8.63)	32.2 (9.9)	37.27 (19.06)	24.2 (6.65)	33.19 (17.12)	NA (NA)	56.0 (37.32)	56.0 (37.32)	31.82 (12.27)	29.09 (12.93)	30.57 (12.6)
total	29.7 (9.53)	29.46 (9.94)	29.59 (9.63)	29.73 (11.46)	27.13 (9.97)	28.4 (10.69)	35.36 (12.87)	32.31 (8.65)	34.17 (11.39)	35.0 (16.02)	29.78 (9.88)	33.19 (14.21)	49.25 (8.96)	54.5 (24.48)	52.75 (20.24)	32.77 (12.59)	31.84 (13.86)	32.36 (13.14)
**Trail Making Test - Part-B**																		
≤ 12 education years	67.0 (18.12)	80.83 (38.03)	73.92 (29.31)	74.12 (33.27)	69.67 (19.48)	72.21 (27.33)	90.27 (23.32)	101.8 (63.61)	93.88 (38.37)	66.83 (32.41)	104.5 (47.54)	81.9 (41.42)	137.75 (84.22)	126.0 (25.43)	131.22 (54.97)	84.0 (41.17)	94.62 (42.49)	88.52 (41.72)
> 12 education years	58.93 (26.75)	64.4 (25.73)	61.26 (26.18)	59.79 (13.27)	65.71 (33.25)	63.03 (25.98)	87.15 (56.85)	65.82 (16.33)	77.38 (43.82)	70.91 (32.57)	47.6 (18.49)	63.62 (30.38)	NA (NA)	68.67 (23.86)	68.67 (23.86)	66.78 (34.96)	63.8 (25.91)	65.4 (31.01)
total	60.39 (25.35)	68.19 (29.02)	63.83 (27.07)	65.0 (22.97)	66.74 (29.89)	65.89 (26.44)	88.58 (43.87)	77.06 (39.41)	83.97 (42.02)	69.47 (31.55)	72.89 (43.79)	70.65 (35.4)	137.75 (84.22)	104.5 (37.58)	115.58 (55.69)	72.81 (37.95)	73.57 (34.95)	73.15 (36.53)
**Letter Fluency Test Total (the sum of all three letters)**																		
≤ 12 education years	37.17 (12.02)	26.4 (8.71)	32.27 (11.59)	38 (10.98)	32.33 (6.15)	35.57 (9.38)	31.27 (10.85)	29.25 (3.86)	30.73 (9.39)	46.83 (6.01)	33.25 (7.93)	41.4 (9.5)	26.75 (9.74)	20.75 (10.97)	23.75 (10.12)	35.97 (11.56)	28.65 (8.34)	33.07 (10.93)
> 12 education years	41.52 (10.8)	36.72 (13.82)	39.6 (12.18)	46.21 (11.98)	41.44 (13.61)	43.67 (12.88)	35.47 (14.62)	43.45 (12.18)	38.85 (13.97)	38.62 (8.03)	39.6 (7.09)	39.0 (7.39)	NA (NA)	36.0 (10.54)	36.0 (10.54)	40.77 (12.1)	39.77 (12.66)	40.32 (12.32)
total	40.73 (10.96)	34.48 (13.43)	38.16 (12.32)	43.23 (12.06)	38.95 (12.59)	41.09 (12.37)	33.69 (13.09)	39.67 (12.31)	35.88 (12.98)	42.14 (8.15)	36.78 (7.74)	40.04 (8.26)	26.75 (9.74)	27.29 (12.79)	27.09 (11.26)	39.07 (12.08)	36.41 (12.57)	37.91 (12.33)
**Letter Fluency Test - 1. Letter**																		
≤ 12 education years	16.33 (4.63)	8.6 (2.51)	12.82 (5.44)	13.88 (4.32)	12.67 (2.8)	13.36 (3.67)	11.27 (4.08)	11.75 (3.5)	11.4 (3.81)	16.5 (2.17)	12.0 (3.56)	14.7 (3.5)	8.25 (4.19)	7.5 (1.0)	7.88 (2.85)	13.29 (4.69)	10.61 (3.3)	12.22 (4.37)
> 12 education years	15.19 (4.63)	13.33 (5.65)	14.44 (5.08)	16.5 (5.13)	14.75 (5.78)	15.57 (5.46)	12.33 (5.37)	14.45 (5.24)	13.23 (5.32)	14.5 (4.11)	15.6 (2.51)	14.92 (3.5)	NA (NA)	13.33 (6.81)	13.33 (6.81)	14.72 (4.97)	14.21 (5.31)	14.49 (5.11)
total	15.39 (4.58)	12.3 (5.46)	14.12 (5.15)	15.55 (4.92)	14.18 (5.16)	14.86 (5.03)	11.88 (4.8)	13.73 (4.88)	12.56 (4.85)	15.36 (3.46)	14.0 (3.39)	14.83 (3.42)	8.25 (4.19)	10.0 (5.07)	9.36 (4.63)	14.21 (4.9)	13.12 (5.05)	13.74 (4.98)
**Letter Fluency Test - 2. Letter**																		

≤ 12 education years	12.33 (5.09)	10.4 (3.05)	11.45 (4.2)	14.38 (4.57)	11.83 (4.17)	13.29 (4.43)	10.82 (4.49)	11.75 (1.5)	11.07 (3.88)	16.33 (2.88)	12.75 (4.19)	14.9 (3.73)	10.5 (3.11)	6.75 (5.56)	8.62 (4.63)	12.8 (4.56)	10.78 (4.09)	12.0 (4.46)
> 12 education years	15.11 (3.63)	13.61 (5.75)	14.51 (4.6)	16.57 (4.11)	14.69 (5.46)	15.57 (4.89)	13.67 (5.29)	16.27 (5.12)	14.77 (5.28)	13.5 (2.78)	12.8 (4.66)	13.23 (3.44)	NA (NA)	11.33 (4.16)	11.33 (4.16)	14.89 (4.15)	14.28 (5.34)	14.62 (4.72)
total	14.61 (3.99)	12.91 (5.39)	13.91 (4.65)	15.77 (4.31)	13.91 (5.21)	14.84 (4.82)	12.46 (5.08)	15.07 (4.85)	13.41 (5.09)	14.71 (3.07)	12.78 (4.18)	13.96 (3.59)	10.5 (3.11)	8.71 (5.22)	9.36 (4.48)	14.15 (4.39)	13.22 (5.23)	13.75 (4.78)
**Letter Fluency Test - 3. Letter**																		
≤ 12 education years	8.5 (3.94)	7.4 (4.28)	8.0 (3.92)	9.75 (2.82)	7.83 (3.37)	8.93 (3.1)	9.18 (3.37)	5.75 (1.71)	8.27 (3.35)	14.0 (3.03)	8.5 (1.73)	11.8 (3.77)	8.0 (4.08)	6.5 (4.8)	7.25 (4.2)	9.89 (3.74)	7.26 (3.28)	8.84 (3.76)
> 12 education years	11.22 (4.44)	9.78 (4.56)	10.64 (4.49)	13.14 (4.69)	12.0 (3.93)	12.53 (4.26)	9.47 (5.03)	12.73 (3.04)	10.85 (4.53)	10.62 (3.29)	11.2 (3.11)	10.85 (3.11)	NA (NA)	11.33 (6.51)	11.33 (6.51)	11.16 (4.59)	11.28 (4.1)	11.21 (4.36)
total	10.73 (4.42)	9.26 (4.51)	10.12 (4.48)	11.91 (4.36)	10.86 (4.17)	11.39 (4.25)	9.35 (4.33)	10.87 (4.17)	9.9 (4.28)	12.07 (3.52)	10.0 (2.83)	11.26 (3.36)	8.0 (4.08)	8.57 (5.68)	8.36 (4.95)	10.71 (4.33)	10.07 (4.27)	10.43 (4.31)

Note: Symbol Digit Modalities Test (min.-max.: 0–110 points in 90 sec); Category fluency (number of correct words - animals in 60 sec); Stroop in each test (min.-max.: 0–200 in 45 sec); Trail Making Test, Part A and Part B (both time in sec, max. 240 sec, the longer time elapsed, the worse performance); Letter fluency test (number of correct words for three letters in 60 sec each, i.e. in total 180 sec), NaN = no participant was available for this cell, NA = the cell is derived from a single participant.

Supplementary Material Table 9

Means and standard deviations for each cognitive test for the Polish sample stratified by age, education, and gender

	Age Group																	
	18-34 years			35-44 years			45-54 years			55-64 years			> 64 years			Total		
	female	male	all	female	male	all	female	male	all	female	male	all	female	male	all	female	male	all
**Symbol Digit Modalities Test**																		
≤ 12 education years	50.2 (4.6)	48.33 (5.92)	49.18 (5.19)	NaN (NA)	30.0 (NA)	30.0 (NA)	40.25 (14.03)	NaN (NA)	40.25 (14.03)	33.0 (2.83)	NaN (NA)	33.0 (2.83)	15.0 (NA)	NaN (NA)	15.0 (NA)	41.08 (13.19)	45.71 (8.79)	42.79 (11.72)
> 12 education years	56.52 (8.68)	53.76 (6.46)	55.29 (7.79)	51.84 (9.78)	42.0 (11.18)	49.19 (10.89)	47.0 (4.58)	47.33 (3.79)	47.1 (4.15)	50.0 (NA)	47.0 (4.24)	48.0 (3.46)	NaN (NA)	51.0 (19.8)	51.0 (19.8)	53.15 (9.11)	49.87 (9.28)	51.86 (9.26)
total	55.31 (8.37)	52.35 (6.65)	53.92 (7.68)	51.84 (9.78)	40.5 (11.19)	48.48 (11.3)	44.55 (9.13)	47.33 (3.79)	45.14 (8.23)	38.67 (10.02)	47.0 (4.24)	42.0 (8.69)	15.0 (NA)	51.0 (19.8)	39.0 (25.06)	50.73 (11.05)	49.11 (9.22)	50.1 (10.36)
**Categorical Fluency Test (Animals)**																		
≤ 12 education years	21.8 (4.44)	23.0 (3.58)	22.45 (3.83)	NaN (NA)	19.0 (NA)	19.0 (NA)	19.5 (3.11)	NaN (NA)	19.5 (3.11)	23.0 (0.0)	NaN (NA)	23.0 (0)	9.0 (NA)	NaN (NA)	9.0 (NA)	20.17 (4.9)	22.43 (3.6)	21.0 (4.5)
> 12 education years	23.05 (5.93)	24.29 (4.57)	23.61 (5.33)	23.16 (4.29)	21.43 (7.7)	22.69 (5.3)	21.17 (5.98)	24.67 (7.57)	22.33 (6.3)	13.0 (NA)	25.5 (0.71)	21.33 (7.23)	NaN (NA)	20.0 (5.66)	20.0 (5.66)	22.64 (5.37)	23.48 (5.52)	22.97 (5.41)
total	22.81 (5.61)	23.96 (4.29)	23.35 (5.02)	23.16 (4.29)	21.12 (7.18)	22.56 (5.24)	20.5 (4.88)	24.67 (7.57)	21.46 (5.55)	19.67 (5.77)	25.5 (0.71)	22.0 (5.2)	9.0 (NA)	20.0 (5.66)	16.33 (7.51)	22.14 (5.33)	23.29 (5.19)	22.59 (5.28)
**Stroop Color Naming Test**																		
≤ 12 education years	67.8 (4.09)	74.83 (7.63)	71.64 (7.02)	NaN (NA)	7.01 (NA)	71.0 (NA)	64.25 (21.33)	NaN (NA)	64.25 (21.33)	76.0 (1.41)	NaN (NA)	76.0 (1.41)	40.0 (NA)	NaN (NA)	40.0 (NA)	65.67 (14.57)	74.29 (7.11)	68.84 (12.84)
> 12 education years	74.33 (11)	77.24 (8.92)	75.63 (10.1)	76.0 (9.12)	62.0 (9.3)	72.64 (10.84)	68.86 (13.35)	69.67 (11.02)	69.1 (12.08)	59.0 (NA)	67.5 (10.61)	64.67 (8.96)	NaN (NA)	59.5 (16.26)	59.5 (16.26)	73.88 (10.79)	71.6 (11.38)	73.0 (11.0)
total	73.08 (10.31)	76.61 (8.5)	74.73 (9.57)	76.0 (9.12)	63.29 (9.14)	72.58 (10.63)	67.18 (15.77)	69.67 (11.02)	67.71 (14.53)	70.33 (9.87)	67.5 (10.61)	69.2 (8.9)	40.0 (NA)	59.5 (16.26)	53.0 (16.09)	72.23 (11.97)	72.11 (10.67)	72.19 (11.44)
**Stroop Word Reading Test**																		
≤ 12 education years	85.8 (8.01)	93.67 (4.13)	90.09 (7.15)	NaN (NA)	86.0 (NA)	86.0 (NA)	79.25 (10.24)	NaN (NA)	79.25 (10.24)	84.5 (7.78)	NaN (NA)	84.5 (7.78)	42.0 (NA)	NaN (NA)	42.0 (NA)	79.75 (14.42)	92.57 (4.76)	84.47 (13.23)
> 12 education years	95.19 (8.38)	93.94 (8.6)	94.63 (8.39)	93.63 (7.24)	81.0 (11.24)	90.6 (9.79)	90.57 (15.84)	96.0 (6.93)	92.2 (13.6)	70.0 (NA)	92.5 (0.71)	85.0 (13.0)	NaN (NA)	65.0 (35.36)	65.0 (35.36)	93.38 (9.81)	89.53 (13.49)	91.9 (11.44)
total	93.38 (8.98)	93.87 (7.59)	93.61 (8.28)	93.63 (7.24)	81.71 (10.44)	90.42 (9.64)	86.45 (14.65)	96.0 (6.93)	88.5 (13.75)	79.67 (10.02)	92.5 (0.71)	84.8 (9.98)	42.0 (NA)	65.0 (35.36)	57.33 (28.31)	90.65 (12.07)	90.11 (12.32)	90.44 (12.1)

**Stroop Interference Test**																		
≤ 12 education years	39.0 (6.12)	45.5 (8.83)	42.55 (8.09)	NaN (NA)	34.0 (NA)	34.9 (NA)	35.25 (12.53)	NaN (NA)	35.25 (12.53)	38.0 (0.0)	NaN (NA)	38.0 (0.0)	15.0 (NA)	NaN (NA)	15.0 (NA)	35.58 (10.07)	43.86 (9.15)	38.63 (10.33)
> 12 education years	43.95 (12.07)	46.76 (7.76)	45.21 (10.34)	42.26 (9.1)	37.33 (7.81)	41.08 (8.91)	41.43 (8.87)	41.0 (9.64)	41.3 (8.55)	NaN (NA)	39.5 (4.95)	39.5 (4.95)	NaN (NA)	39.5 (13.44)	39.5 (13.44)	42.89 (10.34)	43.33 (8.61)	43.06 (9.65)
total	43.0 (11.25)	46.43 (7.86)	44.61 (9.86)	42.26 (9.1)	36.86 (7.24)	40.81 (8.84)	39.18 (10.2)	41.0 (9.64)	39.57 (9.74)	38.0 (0.0)	39.5 (4.95)	38.75 (2.99)	15.0 (NA)	39.5 (13.44)	31.33 (17.04)	41.41 (10.63)	43.43 (8.59)	42.19 (9.89)
**Trail Making Test - Part-A**																		
≤ 12 education years	28.4 (7.7)	22.33 (5.85)	25.09 (7.13)	NaN (NA)	28.0 (NA)	28.0 (NA)	29.25 (4.5)	NaN (NA)	29.25 (4.5)	36.5 (6.36)	NaN (NA)	36.5 (6.36)	111.0 (NA)	NaN (NA)	111.0 (NA)	36.92 (24.17)	23.14 (5.76)	31.84 (20.36)
> 12 education years	25.48 (7.68)	27.24 (9.29)	26.26 (8.37)	27.42 (10.75)	30.14 (9.65)	28.15 (10.34)	33.14 (4.3)	27.5 (12.02)	31.89 (6.17)	45.0 (NA)	33.0 (14.14)	37.0 (12.17)	NaN (NA)	39.5 (10.61)	39.5 (10.61)	27.77 (9.2)	29.13 (9.68)	28.29 (9.35)
total	26.04 (7.62)	25.96 (8.68)	26.0 (8.05)	27.42 (10.75)	29.88 (8.97)	28.15 (10.14)	31.73 (4.58)	27.5 (12.02)	31.08 (5.66)	39.33 (6.66)	33.0 (14.14)	36.8 (9.18)	111.0 (NA)	39.5 (10.61)	63.33 (41.96)	29.6 (13.78)	28.0 (9.31)	28.99 (12.24)
**Trail Making Test - Part-B**																		
≤ 12 education years	81.6 (38.84)	46.33 (9.63)	62.36 (31.45)	NaN (NA)	58.0 (NA)	58.0 (NA)	78.25 (40.17)	NaN (NA)	78.25 (40.17)	81.0 (15.56)	NaN (NA)	81.0 (15.56)	218.0 (NA)	NaN (NA)	218.0 (NA)	91.75 (50.93)	48.0 (9.83)	75.63 (45.69)
> 12 education years	60.35 (27.91)	50.06 (18.46)	55.62 (24.28)	47.37 (14.19)	71.29 (37.36)	53.81 (24.44)	75.14 (37.78)	62.5 (13.44)	72.33 (33.53)	59.0 (NA)	64.0 (21.21)	62.33 (15.28)	NaN (NA)	49.5 (20.51)	49.5 (20.51)	57.28 (26.08)	56.73 (24.49)	57.06 (25.31)
total	64.6 (30.71)	49.09 (16.48)	57.17 (25.89)	47.37 (14.19)	69.62 (34.91)	53.96 (23.98)	76.27 (36.64)	62.5 (13.44)	74.15 (34.07)	73.67 (16.8)	64.0 (21.21)	69.8 (16.78)	218 (NA)	49.5 (20.51)	105.67 (98.36)	64.29 (35.03)	55.08 (22.61)	60.74 (31.04)
**Letter Fluency Test Total (the sum of all three letters)**																		
≤ 12 education years	44.8 (3.27)	39.83 (6.94)	42.09 (5.92)	NaN (NA)	40.0 (NA)	40.0 (NA)	44.25 (6.5)	NaN (NA)	44.25 (6.5)	56.5 (2.12)	NaN (NA)	56.5 (2.12)	16.0 (NA)	NaN (NA)	16.0 (NA)	44.17 (10.76)	39.86 (6.34)	42.58 (9.42)
> 12 education years	45.76 (10.33)	45.35 (9.97)	45.58 (10.04)	48.58 (12.54)	38.14 (14.78)	45.77 (13.71)	49.14 (5.55)	48.0 (19.92)	48.8 (10.44)	29.0 (NA)	42.5 (0.71)	38.0 (7.81)	NaN (NA)	32.0 (15.56)	32 (15.56)	47.02 (10.9)	42.94 (12.27)	45.42 (11.56)
total	45.58 (9.34)	43.91 (9.45)	44.8 (9.33)	48.58 (12.54)	38.38 (13.7)	45.56 (13.49)	47.36 (6.1)	48.0 (19.92)	47.5 (9.48)	47.33 (15.95)	42.5 (0.71)	45.4 (11.59)	16.0 (NA)	32.0 (15.56)	26.67 (14.36)	46.45 (10.85)	42.37 (11.4)	44.87 (11.19)
**Letter Fluency Test - 1. Letter**																		
≤ 12 education years	14.2 (1.64)	12.83 (4.45)	13.45 (3.39)	NaN (NA)	12.0 (NA)	12.0 (NA)	12.25 (2.5)	NaN (NA)	12.25 (2.5)	13.5 (0.71)	NaN (NA)	13.5 (0.71)	3.0 (NA)	NaN (NA)	3.0 (NA)	12.5 (3.53)	12.71 (4.07)	12.58 (3.63)
> 12 education years	13.81 (4.13)	13.94 (3.45)	13.87 (3.79)	14.0 (4.9)	10.57 (3.82)	13.08 (4.82)	15.14 (3.18)	14.67 (4.93)	15.0 (3.5)	7.0 (NA)	13.5 (0.71)	11.33 (3.79)	NaN (NA)	11.5 (2.12)	11.5 (2.12)	13.94 (4.36)	13.06 (3.66)	13.59 (4.1)
total	13.88 (3.76)	13.65 (3.66)	13.78 (3.68)	14.0 (4.9)	10.75 (3.58)	13.04 (4.73)	14.09 (3.18)	14.67 (4.93)	14.21 (3.4)	11.33 (3.79)	13.5 (0.71)	12.2 (2.95)	3.0 (NA)	11.5 (2.12)	8.67 (5.13)	13.65 (4.22)	13.0 (3.68)	13.4 (4.01)
**Letter Fluency Test - 2. Letter**																		

≤ 12 education years	15.0 (1.87)	14.17 (3.13)	14.55 (2.54)	NaN (NA)	14.0 (NA)	14.0 (NA)	16.5 (3.0)	NaN (NA)	16.5 (3.0)	25.0 (0.0)	NaN (NA)	25.0 (0.0)	7.0 (NA)	NaN (NA)	7.0 (NA)	16.5 (5.11)	14.14 (2.85)	15.63 (4.47)
> 12 education years	17.19 (3.86)	15.53 (3.91)	16.45 (3.92)	18.05 (3.78)	14.14 (7.38)	17.0 (5.15)	17.57 (2.7)	18.0 (4.36)	17.7 (3.02)	11.0 (NA)	15.0 (0.0)	13.67 (2.31)	NaN (NA)	10.0 (5.66)	10.0 (5.66)	17.46 (3.71)	15.06 (4.93)	16.52 (4.36)
total	16.77 (3.64)	15.17 (3.7)	16.02 (3.72)	18.05 (3.78)	14.12 (6.83)	16.89 (5.08)	17.18 (2.71)	18.0 (4.36)	17.36 (2.95)	20.33 (8.08)	15.0 (0.0)	18.2 (6.42)	7.0 (NA)	10 (5.66)	9.0 (4.36)	17.27 (4.0)	14.89 (4.6)	16.35 (4.37)
**Letter Fluency Test - 3. Letter**																		
≤ 12 education years	15.6 (1.52)	12.83 (1.72)	14.09 (2.12)	NaN (NA)	14.0 (NA)	14.0 (NA)	15.5 (3.11)	NaN (NA)	15.5 (3.11)	18.0 (2.83)	NaN (NA)	18.0 (2.83)	6.0 (NA)	NaN (NA)	6.0 (NA)	15.17 (3.66)	13.0 (1.63)	14.37 (3.2)
> 12 education years	14.76 (4.25)	15.88 (3.95)	15.26 (4.1)	16.53 (5.35)	13.43 (4.5)	15.69 (5.24)	16.43 (2.3)	15.33 (10.69)	16.1 (5.4)	11.0 (NA)	14.0 (1.41)	13.0 (2.0)	NaN (NA)	10.5 (7.78)	10.5 (7.78)	15.62 (4.53)	14.81 (4.95)	15.3 (4.69)
total	14.92 (3.87)	15.09 (3.73)	15.0 (3.76)	16.53 (5.35)	13.5 (4.17)	15.63 (5.15)	16.09 (2.51)	15.33 (10.69)	15.93 (4.75)	15.67 (4.51)	14.0 (1.41)	15.0 (3.39)	6.0 (NA)	10.5 (7.78)	9.0 (6.08)	15.53 (4.35)	14.47 (4.56)	15.12 (4.44)

Note: Symbol Digit Modalities Test (min.-max.: 0–110 points in 90 sec); Category fluency (number of correct words - animals in 60 sec); Stroop in each test (min.-max.: 0–200 in 45 sec); Trail Making Test, Part A and Part B (both time in sec, max. 240 sec, the longer time elapsed, the worse performance); Letter fluency test (number of correct words for three letters in 60 sec each, i.e. in total 180 sec), NaN = no participant was available for this cell, NA = the cell is derived from a single participant

Supplementary Material Table 10

Means and standard deviations for each cognitive test for the Canadian French sample stratified by age, education, and gender

	Age Group																	
	18-34 years			35-44 years			45-54 years			55-64 years			> 64 years			Total		
	female	male	all	female	male	all	female	male	all	female	male	all	female	male	all	female	male	all
**Symbol Digit Modalities Test**																		
≤ 12 education years	54.67 (12.37)	44.5 (15.37)	50.6 (13.83)	50.5 (2.12)	NaN (NA)	50.5 (2.12)	49.0 (9.66)	49.4 (9.74)	49.22 (9.08)	44.0 (11.69)	31.67 (9.07)	38.71 (11.8)	40.67 (9.07)	37.0 (NA)	39.75 (7.63)	48.58 (10.87)	42.85 (12.54)	46.25 (11.73)
> 12 education years	61.5 (9.57)	65.4 (13.09)	63.27 (10.88)	58.38 (6.5)	73.0 (NA)	60.0 (7.79)	54.12 (7.41)	50.5 (3.54)	53.4 (6.82)	49.0 (5.0)	46.0 (4.58)	47.5 (4.59)	46.0 (NA)	42.0 (NA)	44.0 (2.83)	56.23 (8.22)	56.75 (13.55)	56.39 (10.01)
total	58.08 (11.13)	56.11 (17.19)	57.24 (13.69)	56.8 (6.66)	73.0 (NA)	58.27 (7.99)	52.42 (8.17)	49.71 (8.1)	51.42 (8.03)	46.14 (9.15)	38.83 (10.15)	42.77 (9.96)	42.0 (7.87)	39.5 (3.54)	41.17 (6.43)	53.0 (10.07)	49.52 (14.6)	51.76 (11.9)
**Categorical Fluency Test (Animals)**																		
≤ 12 education years	18.83 (5.23)	14.75 (7.68)	17.2 (6.27)	23.5 (3.54)	NaN (NA)	23.5 (3.54)	18.25 (8.62)	19.6 (1.67)	19.0 (5.45)	20.25 (2.06)	13.0 (1.73)	17.14 (4.26)	14.0 (2.65)	12.0 (NA)	13.5 (2.38)	18.74 (5.38)	16.0 (5.07)	17.62 (5.35)
> 12 education years	22.5 (3.02)	21.4 (4.83)	22.0 (3.77)	23.12 (5.49)	30.0 (NA)	23.89 (5.62)	21.62 (3.58)	22.5 (3.54)	21.8 (3.39)	24.67 (4.16)	17.67 (4.51)	21.17 (5.46)	24.0 (NA)	19.0 (NA)	21.5 (3.54)	22.73 (4.03)	21.17 (4.95)	22.24 (4.33)
total	20.67 (4.5)	18.44 (6.78)	19.71 (5.55)	23.2 (4.98)	30.0 (NA)	23.82 (5.15)	20.5 (5.58)	20.43 (2.44)	20.47 (4.59)	22.14 (3.67)	15.33 (3.98)	19.0 (5.08)	16.5 (5.45)	15.5 (4.95)	16.17 (4.79)	21.04 (5.0)	18.48 (5.57)	20.13 (5.32)
**Stroop Color Naming Test**																		
≤ 12 education years	74.83 (13.82)	66.5 (15.93)	71.5 (14.46)	77.0 (12.73)	NaN (NA)	77.0 (12.73)	73.5 (16.46)	85.2 (8.11)	80.0 (13.13)	63.75 (18.46)	63.67 (8.08)	63.71 (13.86)	70.33 (11.5)	65.0 (NA)	69.0 (9.76)	71.74 (14.15)	72.92 (14.12)	72.22 (13.92)
> 12 education years	89.83 (12.43)	87.8 (10.57)	88.91 (11.09)	84.12 (6.42)	89.0 (NA)	84.67 (6.22)	80.0 (13.31)	80.0 (11.31)	80.0 (12.33)	82.33 (6.43)	72.67 (17.01)	77.5 (12.66)	72.0 (NA)	63.0 (NA)	67.5 (6.36)	83.5 (10.7)	80.75 (13.42)	82.63 (11.52)
total	82.33 (14.78)	78.33 (16.64)	80.62 (15.33)	82.7 (7.69)	89.0 (NA)	83.27 (7.54)	77.83 (14.03)	83.71 (8.46)	80.0 (12.36)	71.71 (16.82)	68.17 (12.89)	70.08 (14.63)	70.75 (9.43)	64.0 (1.41)	68.5 (8.12)	78.53 (13.47)	76.68 (14.08)	77.87 (13.62)
**Stroop Word Reading Test**																		
≤ 12 education years	95.83 (14.47)	77.25 (29.55)	88.4 (22.35)	93.0 (12.73)	NaN (NA)	93.0 (12.73)	96.0 (19.58)	91.4 (30.56)	93.44 (24.83)	87.25 (17.23)	69.67 (22.01)	79.71 (19.96)	91.0 (7.21)	78.0 (NA)	87.75 (8.77)	93.0 (14.11)	81.0 (26.32)	88.12 (20.48)
> 12 education years	102 (13.33)	114.6 (10.62)	107.73 (13.31)	97.75 (26.02)	100.0 (NA)	98.0 (24.35)	105.75 (14.93)	101 (1.41)	104.8 (13.32)	106.67 (5.03)	87.67 (14.84)	97.17 (14.37)	97.0 (NA)	93.0 (NA)	95.0 (2.83)	102.19 (17.42)	102.58 (14.73)	102.32 (16.42)
total	98.92 (13.65)	98.0 (27.77)	98.52 (20.28)	96.8 (23.42)	100.0 (NA)	97.09 (22.24)	102.5 (16.41)	94.14 (25.39)	99.42 (19.92)	95.57 (16.27)	78.67 (19.47)	87.77 (19.16)	92.5 (6.61)	85.5 (10.61)	90.17 (7.86)	98.31 (16.58)	91.36 (23.81)	95.83 (19.59)

**Stroop Interference Test**																		
≤ 12 education years	48.0 (11.54)	34.75 (9)	42.7 (12.16)	38.0 (2.83)	NaN (NA)	38.0 (2.83)	38.75 (11.12)	46.4 (17.36)	43.0 (14.6)	35.25 (9.54)	33.67 (4.04)	34.57 (7.18)	30.67 (5.51)	35.0 (NA)	31.75 (4.99)	39.58 (10.86)	39.0 (12.68)	39.34 (11.44)
> 12 education years	51.83 (24.41)	54.2 (8.01)	52.91 (18.03)	47.5 (7.07)	50.0 (NA)	47.78 (6.67)	42.12 (8.39)	43.0 (8.49)	42.3 (7.93)	45.0 (10.0)	38.0 (10.82)	41.5 (10.07)	36.0 (NA)	34.0 (NA)	35.0 (1.41)	46.12 (13.37)	46.25 (10.74)	46.16 (12.45)
total	49.92 (18.31)	45.56 (12.94)	48.05 (16.01)	45.6 (7.47)	50.0 (NA)	46.0 (7.21)	41.0 (9.02)	45.43 (14.68)	42.63 (11.24)	39.43 (10.29)	35.83 (7.68)	37.77 (9.0)	32.0 (5.23)	34.5 (0.71)	32.83 (4.26)	43.36 (12.67)	42.48 (12.12)	43.04 (12.39)
**Trail Making Test - Part-A**																		
≤ 12 education years	19.0 (4.0)	24.33 (5.86)	21.29 (5.25)	23.0 (0.0)	NaN (NA)	23.0 (0.0)	19.0 (7.21)	21.5 (6.81)	20.43 (6.5)	34.0 (5.72)	37.0 (12.73)	35.0 (7.38)	40.33 (4.93)	26.0 (NA)	36.75 (8.22)	27.25 (10.02)	25.9 (8.82)	26.73 (9.42)
> 12 education years	19.33 (4.68)	24.8 (14.65)	21.82 (10.25)	19.75 (3.92)	24.0 (NA)	20.22 (3.93)	23.12 (5.77)	20.5 (3.54)	22.6 (5.34)	22.33 (1.53)	30.0 (14.8)	26.17 (10.3)	28.0 (NA)	42.0 (NA)	35.0 (9.9)	21.31 (4.78)	26.75 (12.35)	23.03 (8.21)
total	19.2 (4.18)	24.62 (11.51)	21.61 (8.46)	20.4 (3.72)	24.0 (NA)	20.73 (3.69)	22.0 (6.12)	21.17 (5.53)	21.71 (5.75)	29.0 (7.48)	32.8 (12.83)	30.58 (9.71)	37.25 (7.37)	34.0 (11.31)	36.17 (7.81)	23.57 (7.7)	26.36 (10.65)	24.53 (8.84)
**Trail Making Test - Part-B**																		
≤ 12 education years	37.75 (5.91)	63.67 (44.56)	48.86 (29.52)	47.0 (8.49)	NaN (NA)	47.0 (8.49)	32.0 (13.08)	59.25 (22.41)	47.57 (22.81)	58.5 (6.56)	98.0 (55.15)	71.67 (32.41)	106.33 (13.32)	49.0 (NA)	92.0 (30.66)	55.88 (28.11)	67.3 (35.02)	60.27 (30.78)
> 12 education years	31.67 (5.2)	38.0 (12.63)	34.55 (9.4)	38.25 (10.15)	38.0 (NA)	38.22 (9.5)	42.62 (6.97)	43.0 (7.07)	42.7 (6.58)	52.0 (1.73)	61.33 (6.03)	56.67 (6.47)	58.0 (NA)	66.0 (NA)	62.0 (5.66)	40.42 (9.9)	47.0 (14.32)	42.5 (11.7)
total	34.1 (6.05)	47.62 (28.89)	40.11 (20.27)	40.0 (10.09)	38.0 (NA)	39.82 (9.59)	39.73 (9.63)	53.83 (19.54)	44.71 (15.02)	55.71 (5.88)	76.0 (34.38)	64.17 (23.62)	94.25 (26.5)	57.5 (12.02)	82.0 (28.47)	46.31 (20.16)	56.23 (27.2)	49.72 (23.1)
**Letter Fluency Test Total (the sum of all three letters)**																		
≤ 12 education years	29.5 (3.11)	19.67 (8.5)	25.29 (7.52)	34.0 (9.9)	NaN (NA)	34.0 (9.9)	46.67 (10.02)	45.5 (9.29)	46.0 (8.77)	38.25 (9.6)	22.0 (9.9)	32.83 (12.06)	41.0 (6.24)	54.0 (NA)	44.25 (8.26)	37.62 (9.14)	33.9 (16.11)	36.19 (12.12)
> 12 education years	48.17 (13.14)	40.0 (9.27)	44.45 (11.78)	50.5 (13.02)	47.0 (NA)	50.11 (12.23)	44.5 (13.24)	57.5 (27.58)	47.1 (15.84)	47.33 (14.5)	30.67 (8.02)	39.0 (13.9)	41.0 (NA)	45.0 (NA)	43.0 (2.83)	47.38 (12.47)	41.58 (13.98)	45.55 (13.06)
total	40.7 (13.86)	32.38 (13.44)	37.0 (13.93)	47.2 (13.82)	47.0 (NA)	47.18 (13.11)	45.09 (12)	49.5 (15.57)	46.65 (13.05)	42.14 (11.82)	27.2 (8.9)	35.92 (12.82)	41.0 (5.1)	49.5 (6.36)	43.83 (6.55)	43.67 (12.18)	38.09 (15.13)	41.75 (13.42)
**Letter Fluency Test - 1. Letter**																		
≤ 12 education years	13.0 (1.15)	6.67 (3.51)	10.29 (4.03)	12.5 (3.54)	NaN (NA)	12.5 (3.54)	17.33 (5.13)	17.0 (3.74)	17.14 (3.98)	16.25 (3.86)	10.5 (4.95)	14.33 (4.76)	17.0 (2.65)	21.0 (NA)	18.0 (2.94)	15.31 (3.55)	13.0 (6.25)	14.42 (4.79)
> 12 education years	18.33 (5.05)	15.2 (2.86)	16.91 (4.32)	19.5 (5.88)	24.0 (NA)	20.0 (5.7)	16.12 (5.36)	21.0 (7.07)	17.1 (5.67)	17.67 (9.07)	12.33 (4.73)	15.0 (7.1)	13.0 (NA)	17.0 (NA)	15.0 (2.83)	17.73 (5.67)	16.33 (5.09)	17.29 (5.47)
total	16.2 (4.71)	12.0 (5.26)	14.33 (5.27)	18.1 (6.08)	24.0 (NA)	18.64 (6.04)	16.45 (5.07)	18.33 (4.76)	17.12 (4.9)	16.86 (5.96)	11.6 (4.28)	14.67 (5.77)	16.0 (2.94)	19.0 (2.83)	17.0 (3.03)	16.81 (5.07)	14.82 (5.76)	16.12 (5.36)
**Letter Fluency Test - 2. Letter**																		

≤ 12 education years	8.75 (1.89)	6.0 (4.36)	7.57 (3.21)	9.5 (4.95)	NaN (NA)	9.5 (4.95)	16.33 (1.53)	15.25 (3.2)	15.71 (2.5)	10.25 (3.4)	6.0 (4.24)	8.83 (3.92)	10.67 (1.15)	19.0 (NA)	12.75 (4.27)	11.0 (3.56)	11.0 (6.22)	11.0 (4.64)
> 12 education years	15.5 (5.13)	14.4 (3.29)	15.0 (4.22)	16.62 (4.96)	13.0 (NA)	16.22 (4.79)	14.5 (5.15)	18.5 (12.02)	15.3 (6.29)	15.33 (8.08)	10.33 (3.51)	12.83 (6.21)	15.0 (NA)	14.0 (NA)	14.5 (0.71)	15.5 (5.05)	13.92 (5.18)	15.0 (5.08)
total	12.8 (5.29)	11.25 (5.52)	12.11 (5.29)	15.2 (5.55)	13.0 (NA)	15.0 (5.31)	15.0 (4.45)	16.33 (6.15)	15.47 (4.96)	12.43 (5.91)	8.6 (4.04)	10.83 (5.37)	11.75 (2.36)	16.5 (3.54)	13.33 (3.44)	13.79 (5.01)	12.59 (5.73)	13.38 (5.25)
**Letter Fluency Test - 3. Letter**																		
≤ 12 education years	7.75 (1.71)	7.0 (1.0)	7.43 (1.4)	12.0 (1.41)	NaN (NA)	12.0 (1.41)	13.0 (3.46)	13.25 (3.3)	13.14 (3.08)	11.75 (6.5)	5.5 (0.71)	9.67 (5.99)	13.33 (4.16)	14.0 (NA)	13.5 (3.42)	11.31 (4.24)	9.9 (4.23)	10.77 (4.21)
> 12 education years	14.33 (4.46)	10.4 (3.91)	12.55 (4.5)	14.38 (3.58)	10.0 (NA)	13.89 (3.66)	13.88 (3.91)	18.0 (8.49)	14.7 (4.79)	14.33 (4.93)	8.0 (1.73)	11.17 (4.79)	13.0 (NA)	14.0 (NA)	13.5 (0.71)	14.15 (3.73)	11.33 (5.0)	13.26 (4.31)
total	11.7 (4.85)	9.12 (3.48)	10.56 (4.38)	13.9 (3.35)	10.0 (NA)	13.55 (3.39)	13.64 (3.64)	14.83 (5.19)	14.06 (4.13)	12.86 (5.58)	7.0 (1.87)	10.42 (5.23)	13.25 (3.4)	14.0 (0.0)	13.5 (2.66)	13.07 (4.12)	10.68 (4.61)	12.25 (4.41)

Note: Symbol Digit Modalities Test (min.-max.: 0–110 points in 90 sec); Category fluency (number of correct words - animals in 60 sec); Stroop in each test (min.-max.: 0–200 in 45 sec); Trail Making Test, Part A and Part B (both time in sec, max. 240 sec, the longer time elapsed, the worse performance); Letter fluency test (number of correct words for three letters in 60 sec each, i.e. in total 180 sec), NaN = no participant was available for this cell, NA = the cell is derived from a single participant

Supplementary Material Figure 1

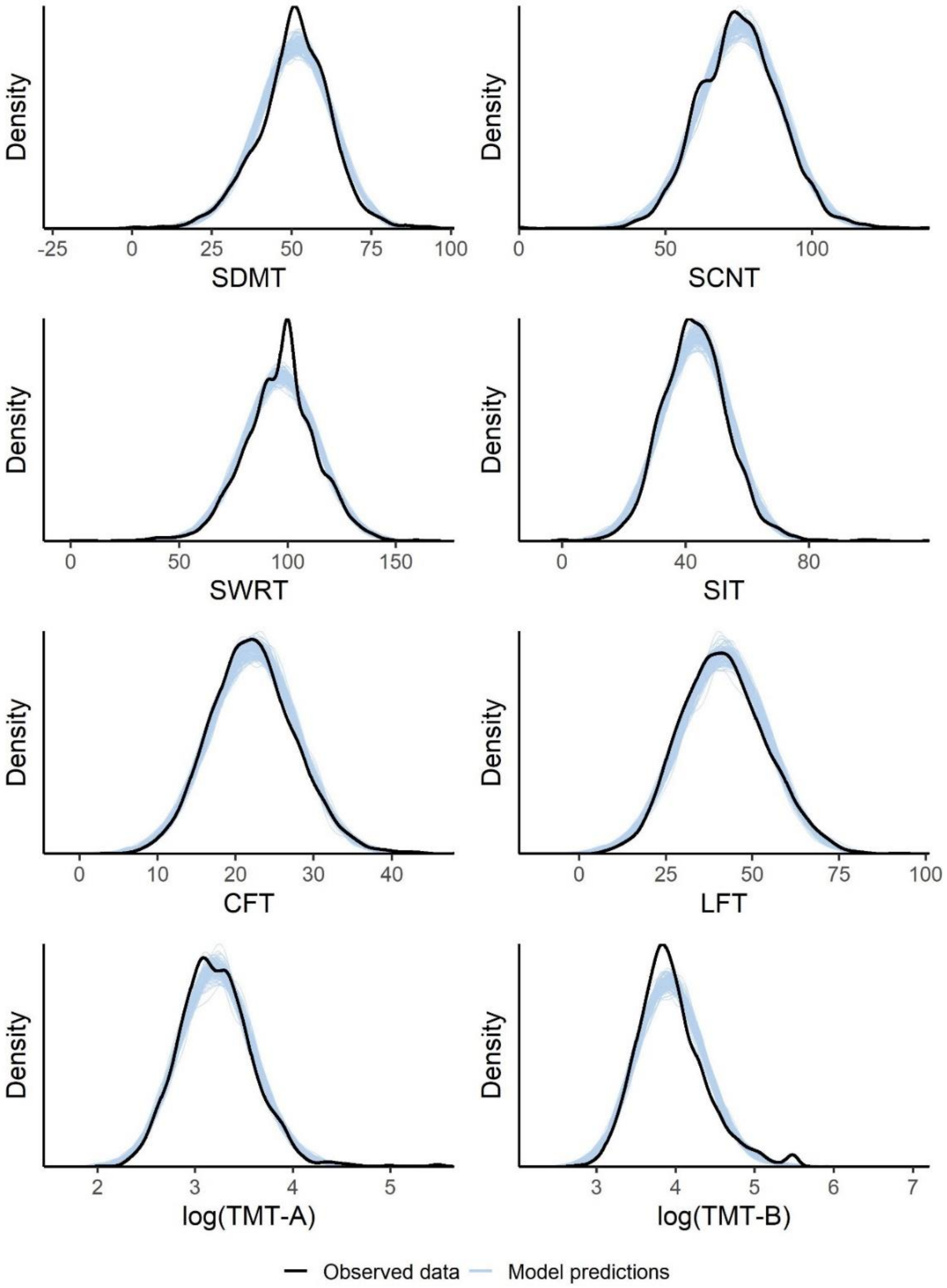

.

Posterior predictive checks of models used to generate normative z-values: SDMT: Symbol Digit Modalities Test, SCNT: Stroop Color Naming Test; SWRT: Stroop Word Reading Test, SIT: Stroop Interference Test, CFT: Category Fluency, VFT: Verbal Fluency Test; TMT-A = Trail Making Test, Part A; TMT-B = Trail Making Test, Part B.

Supplementary Material Figure 2

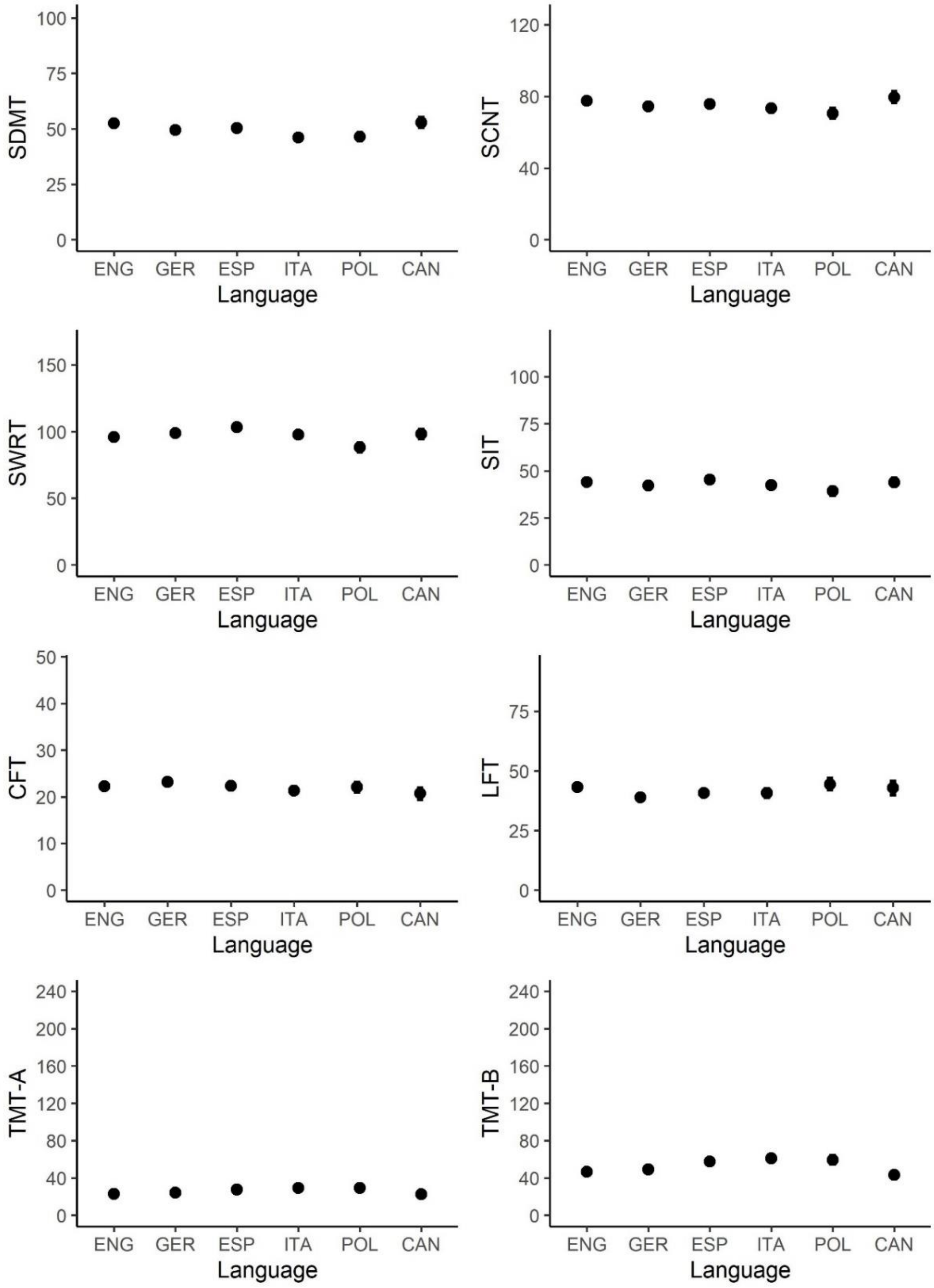

Language differences in tested cognitive measures: Points represent mean estimates of the average performance based on language when age and education are held at median value with respect to the normative sample (i.e., 48 years of age and 14 years of formal education), whiskers estimate 95% posterior probability intervals (PPIs) of the means (in most cases, the mean point covers entire 95% , PPI whiskers).

Supplementary Material Figure 3

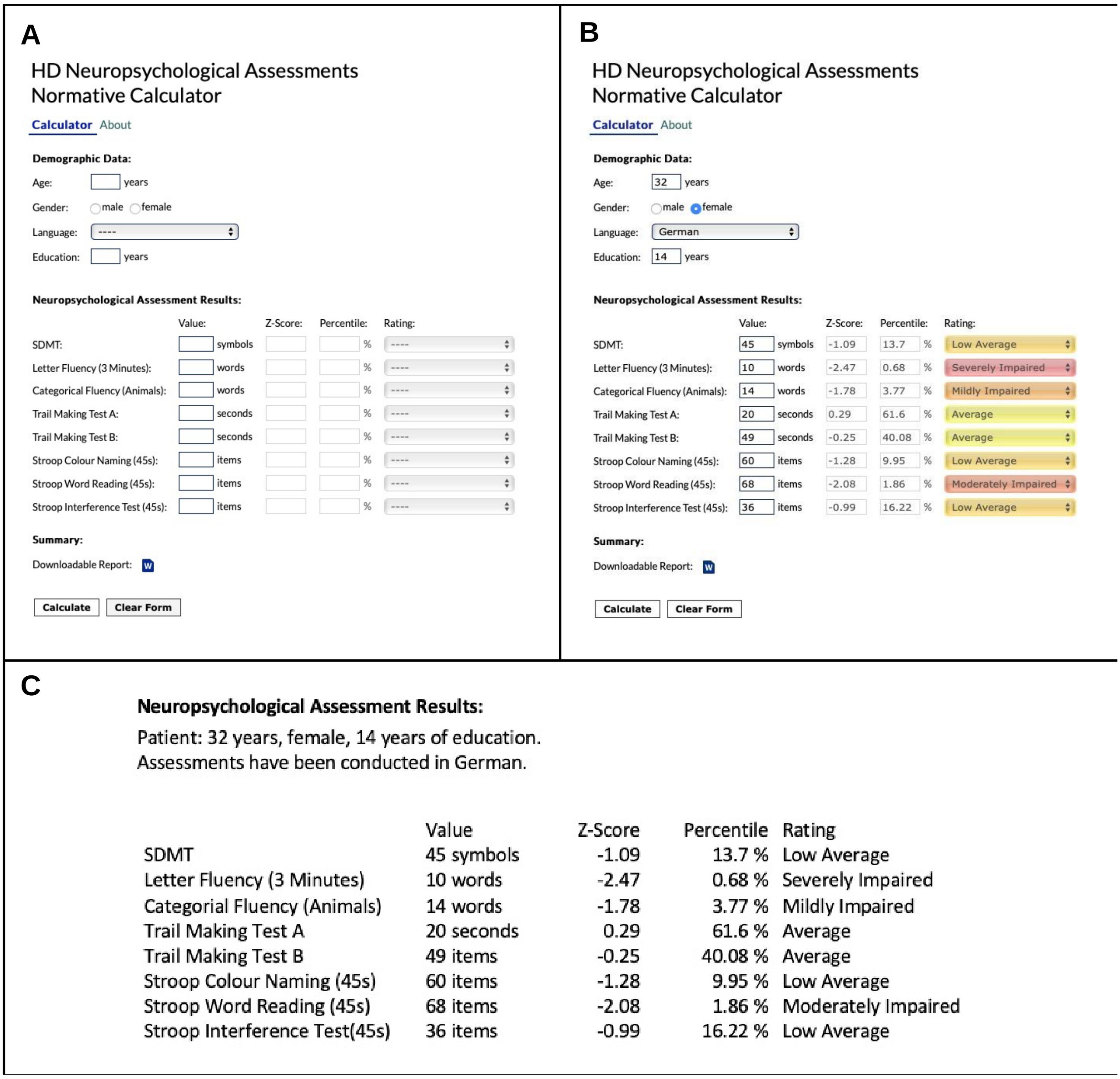

Neuropsychological Assessment Results:

Patient: 32 years, female, 14 years of education.
Assessments have been conducted in German.

	Value	Z-Score	Percentile	Rating
SDMT	45 symbols	-1.09	13.7 %	Low Average
Letter Fluency (3 Minutes)	10 words	-2.47	0.68 %	Severely Impaired
Categorial Fluency (Animals)	14 words	-1.78	3.77 %	Mildly Impaired
Trail Making Test A	20 seconds	0.29	61.6 %	Average
Trail Making Test B	49 items	-0.25	40.08 %	Average
Stroop Colour Naming (45s)	60 items	-1.28	9.95 %	Low Average
Stroop Word Reading (45s)	68 items	-2.08	1.86 %	Moderately Impaired
Stroop Interference Test(45s)	36 items	-0.99	16.22 %	Low Average

The normative calculator: From upper left to right: A) Entry forms (demographics and raw values obtained for each cognitive test) with automatic export function including a downloadable report in RTF format, B) Example output for a 32-year-old HD patient, C) Downloadable report in RTF-format

Supplementary Material Figure 4

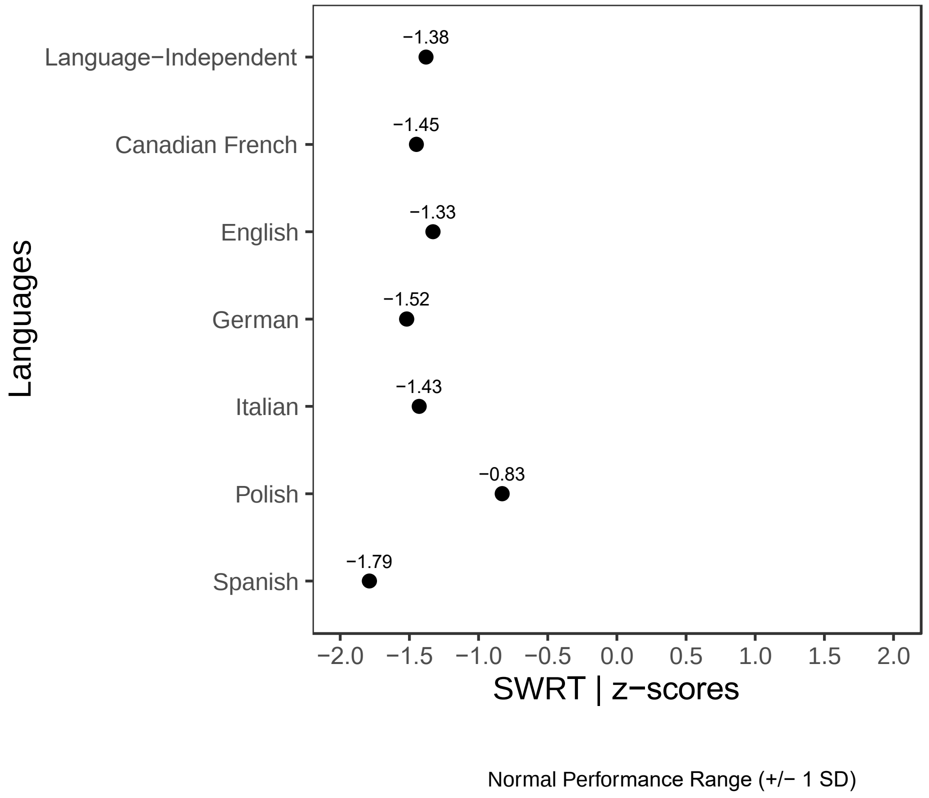

Illustrative Case Study: 32-years old female manifest HD patient, graph showing language-dependent differences in evaluating performance on Stroop Word Reading Test.
